# Context for layering women’s nutrition interventions on a large scale poverty alleviation program: Evidence from three eastern Indian states

**DOI:** 10.1371/journal.pone.0210836

**Published:** 2019-01-22

**Authors:** Reshmi R. S., Konsam Dinachandra, Arti Bhanot, Sayeed Unisa, Gopinath T. Menon, Neeraj Agrawal, Vikas Bhatia, Manisha Ruikar, Abner Daniel, Sourav Bhattacharjee, Rabi N. Parhi, H. P. S. Sachdev, Raj Kumar Gope, Arjan De Wagt, Vani Sethi

**Affiliations:** 1 International Institute for Population Sciences, Mumbai, India; 2 Independent consultant, New Delhi, India; 3 SPARSH-Center for Participatory Learning, Mumbai, India; 4 All India Institute of Medical Sciences (AIIMS), Patna, India; 5 All India Institute of Medical Sciences (AIIMS), Bhubaneswar, India; 6 All India Institute of Medical Sciences (AIIMS), Raipur, India; 7 Nutrition Section, UNICEF India, Country Office, New Delhi, India; 8 UNICEF India, Field Office Odisha, Bhubaneswar, India; 9 UNICEF India, Field Office Bihar, Patna, India; 10 Sitaram Bhartia Institute of Science and Research, New Delhi, India; 11 Ekjut, Jharkhand, India; ESIC Medical College & PGIMSR, INDIA

## Abstract

Over 70 million women of reproductive age are undernourished in India. Most poverty alleviation programs have not been systematically evaluated to assess impact on women’s empowerment and nutrition outcomes. National Rural Livelihoods Mission’s poverty alleviation and livelihoods generation initiative is an opportune platform to layer women’s nutrition interventions being tapped by project Swabhimaan in three eastern Indian states—Bihar, Chhattisgarh and Odisha. A cross-sectional baseline survey covering 8755 mothers of children under-two years of age, one of the three primary target groups of program are presented. Standardized questionnaire was administered and anthropometric measurements were undertaken from October 2016 to January 2017. 21 indicators on women’s empowerment, Body Mass Index and Mid-upper Arm Circumference for nutrition status, food insecurity indicators as per the Food Insecurity Experience Scale and selected indicators for assessing women’s access to basic health services were included. National Rural Livelihoods Mission operates in contexts with stark social and gender inequalities. Self-help group members exhibited better control on financial resources and participation in community activities than non-members. Using Body Mass Index, at least 45% mothers were undernourished irrespective of their enrolment in self-help groups. Higher proportion of self-help group members (77%-87%) belonged to food insecure households than non-members (66%-83%). Proportion of mothers reporting receipt of various components of antenatal care service package varied from over 90% for tetanus toxoid vaccination to less than 10% for height measurement. Current use of family planning methods was excruciatingly low (8.2%-32.4%) in all states but positively skewed towards self-help group members. Participation in monthly fixed day health camps was a concern in Bihar. Layering women’s nutrition interventions as stipulated under Swabhimaan may yield better results for women’s empowerment and nutrition status under National Rural Livelihoods Mission. While this opportunity exists in all three states, Bihar with a higher proportion of matured self-help groups offers more readiness for Swabhimaan implementation.

## Introduction

India had an estimated 71 million undernourished women of reproductive age and an estimated 30 million pregnancies [1, 2]. Majority of these pregnancies were expectedly among undernourished women and a quarter among adolescent girls [3]. The long term, intergenerational and irreversible consequences of pre-pregnancy and maternal undernutrition, manifesting as intra-uterine growth retardation, prematurity, low birth weight and childhood stunting as well as increased susceptibility to chronic diseases later in life are established [4]. A five year initiative titled Swabhimaan (Lit. Pride) was launched in 2016, layering essential women’s nutrition interventions on an ongoing nation-wide government livelihoods promotion and economic empowerment initiative- The National Rural Livelihoods Mission (NRLM). Swabhimaan covers over 600 000 population in three states of eastern India- Bihar, Chhattisgarh and Odisha, with the highest rates of women’s undernutriton and fertility. It covers all the stages of a woman’s life-cycle with hightened nutritional vulnerability that is adolesence, pre-pregnancy (newlyweds), pregnancy and lactation (mothers of children under-two). Its design was informed by a scoping study that concluded that mature women’s Self- Help Groups (SHGs) and their federations also known as Village Organizations (VOs) and Cluster Level Federations (CLFs) have the potential to manage grants for improving last mile delivery of essential nutrition services for women, provided they are enabled, supervised and provided protection against domestic violence and exploitation [5]. The concept of layering nutrition interventions on economic empowerment programs for women is not new in the Indian context with programs like Kudumbashree and Andhra Pradesh Rural Poverty Reduction Program providing nearly two decades of learnings [6, 7]. However, Swabhimaan is a pioneering women’s nutrition focused initiative covering direct or nutrition-specific interventions impacting quality and quantity of food intake and nutrition-sensitive interventions pertaining to planned fertility, access to basic health, water, sanitation and hygiene (WASH) services and improved household food security.

Swabhimaan is completely embedded in the government funded NRLM, using its administrative structures and thrift and credit processes. Launched in 2011, NRLM aims to enroll at least one woman from each household in the poorest administrative regions of the country under SHGs; it has reached over 50 million households nation-wide since inception [8, 9]. Swabhimaan facilitates achieving NRLM’s objectives of economically self-reliant women from poorest households by creating entrepreneurs for women’s nutrition relevant services and products. It aims to increase capacity of VOs in managing nutrition grants and women’s agency to demand services and have access to nutrition safety-nets (Table 1) [10].

**Table 1:** Roles of SHGs, VOs and CLFs under Swabhimaan.

SHG or selected representative (Poshan Sakhi^a^)	VO (1 per 5 to 30 SHGs)	CLF (1 per 3–4 VOs)
Monthly meetings with women using Participatory Learning and Action (PLA)	Selection of Poshan Sakhi	Organize biannual newlywed couples meet
Community mobilization to avail services at monthly fixed-day government health camp^b^	Developing and managing Poshan microplan (1 per VO)	Quarterly training of Poshan Sakhis and Farmer producer group representatives
Become role models for recommended WASH practices	Promote adolescent girls groups	Organize biannual entitlement and check-up camps for SHG members
Promote nutri-gardens, backyard poultry		Link nutritionally “at risk” women and children to nutrition safety nets
		Create farmer field school sites
		Annual community audit of Poshan microplan

^a^Poshan is nutrition promotion and Sakhi is female friend. She is a local SHG member with demonstrated leadership skills and committed to community development.

^b^ also referred to as Village Health Sanitation and Nutrition Day (VHSND)

Mixed results have been reported from evaluations of livelihoods programs and their impact on women’s empowerment and health and nutrition outcomes [11, 12]. This has been partially attributed to the complexity of these programs and use of non-gold standard evaluation designs [11, 12, 13]. Swambhimaan’s evaluation design includes three rounds of surveys- baseline, midline and endline to assess achievement of its impact which is hypothesized as: 1) achieving 15% reduction in the proportion of adolescent girls with a Body Mass Index (BMI) <18.5 kg/m^2^, a 15% reduction in the proportion of mothers of children under two with a BMI <18.5 kg/m^2^, and a 0.4 cm improvement in mean Mid-Upper Arm Circumference (MUAC) among pregnant women and 2) between 5% and 20% increase in coverage of 18 key nutrition specific and sensitive interventions (Table 2). These targets were set based on changes in nutritional status and service coverage among adolescent girls and women between 2005–06 available through Demographic Health Survey and most recent large scale health and nutrition surveys for Bihar, Chhattisgarh and Odisha.

**Table 2:** Nutrition-specific and nutrition-sensitive interventions package, Swabhimaan.

	Relevant target group		
	Preconception	Pregnancy	Lactation
**Improve food and nutrient intake**			
1. Access to generalized household ration through Public Distribution System (PDS), a food subsidy scheme	*	*	*
2. Balanced energy protein supplementation through access to supplementary rations	*	*	*
3. Access to knowledge and choices about how to increase maternal dietary diversity	*	*	*
4. Access to knowledge and support for nutrition-sensitive agriculture at home (kitchen garden) and community based food insecurity coping strategies.	*	*	*
**Prevent micronutrient deficiencies and anemia**			
5. Iron Folic Acid (IFA) supplementation	*	*	*
6. Universal use of iodized salt	*	*	*
7. Calcium supplementation and deworming	NA	*	*
8. Access to information and commodities like insecticide treated bed-nets for malaria prevention	*	*	*
9. Access to information on preventing tobacco and alcohol use in pregnancy	NA	*	*
**Increase access to health services and special care to nutritionally “at risk” women**			
10 Early registration in outreach services	*	*	NA
11. Recording and monitoring of nutritional status and special community-based at-nutritional risk package	NA	*	NA
12. Quality reproductive health, antenatal and postnatal care	*	*	*
13. Access to knowledge and entitlements for promotion of institutional deliveries and maternity benefits	NA	*	*
**Increase access to education and commodities for WASH**			
14. Sanitation and hygiene (including menstrual hygiene) education	*	*	*
15. Access to safe drinking water and sanitation commodities	*	*	*
**Prevent early, poorly spaced or unwanted pregnancies**			
16. Promotion of secondary education and education for delaying the age at marriage to legal age	*	NA	NA
17. Access to information and family planning commodities for delaying age at first pregnancy and prevention of repeated pregnancies	*	*	*
18. Women’s collective voice and empowerment for decision-making to prevent child marriage, violence against women, child spacing and other gender-related issues	*	*	*

* implies applicable, NA Not applicable

It also includes a series of process evaluations including community audits [14]. This paper presents findings on women’s empowerment, nutrition status, household food security and use of maternal health services from baseline survey of Swabhimaan, as the initiative tests the effectiveness of its service delivery model in improving these variables. The paper presents the scenario or context prior to layering of women’s nutrition interventions on NRLM platforms. The midline and endline surveys will be completed in 2019 and 2021, respectively.

## Materials and methods

### Sample

Baseline survey covered adolescent girls, pregnant women and mothers of children under-two. Findings from a sample of 8755 mothers of children under-two from selected blocks of Bihar, Chhattisgarh and Odisha are presented in this paper (Table 3). Sample selection was based on simple random sampling.

**Table 3:** Sampling for all target groups, Swabhimaan.

Target group	Intervention	Control	Total
**Adolescent girls**	**3055**	**3297**	**6352**
Bihar	863	841	1704
Chhattisgarh	1468	1453	2921
Odisha	724	1003	1727
**Pregnant women**	**1277**	**1296**	**2573**
Bihar	468	468	936
Chhattisgarh	442	381	823
Odisha	367	447	814
**Mother of children under 2yrs**	**4441**	**4314**	**8755**
Bihar	1400	1212	2612
Chhattisgarh	1281	1258	2539
Odisha	1760	1844	3604

### Tools

Bilingual questionnaire (Bihar: English and Hindi, Chhattisgarh: English and Hindi and Odisha: English and Odia) for mothers of children under-two consisting of 128 questions and probes was developed, field tested and standardized for all states. Household survey questionnaire and interview schedule used with mothers of children under two are shared as supporting information (Refer S1 Appendix for household survey questionnaire and S2 Appendix for interview schedule). Information obtained included but was not limited to socio-demographic and household characteristics, educational attainment, diet diversity, household food security, access to services. Participation in household decision making, autonomy, domestic violence, ownership of money in terms of cash, bank account, work participation, involvement in organization or participation in community level program, access to resources like mobile phone, house or land were used to assess women’s empowerment. Anthropometric measurements (weight, height and MUAC) were conducted using standard techniques [15]. Weight to the nearest 0.1 kg was recorded using a SECA electronic weighing scale with minimal clothing. Height was taken barefoot to the nearest 0.1 cm using a stadiometer. MUAC was measured to the nearest 0.1 cm with a non-stretchable measuring tape.

### Data collection and timelines

Data collection teams included 30 trained investigators in each state, supervised by six field supervisors and six field editors. Quality control checks were conducted for 10% of the interviewed population. The weighing scales and stadiometer were calibrated on a weekly basis prior to data collection with standard weights (1, 2 and 5 kg) and a metre rod (100 cm). The mean standard errors of measurement for height, weight, and MUAC across all the data collection teams were insignificant and ranged between 0.001‒0.025 (*p* < 0.10, CI = -0.004‒0.042). Data collection was completed between October 2016 and January 2017 across three states. Complete data set is available as supporting information (S3 Appendix).

### Indicators

Women’s empowerment indicators included indicators for assessing women’s agency through participation in household decision making, freedom of mobility outside house and perception of domestic violence issues. It also included her access to resources, leadership opportunities, income generation activities, all measured through 21 indicators. These have also been used by other researchers to assess women’s empowerment [16]. Women’s nutrition indicators included globally accepted indicator of BMI {weight (kg)/ height (m)^2^} with the established cut-off of <18.5 kg/m^2^ to determine underweight, as well as MUAC which is being increasing reported in recent researches as a reliable measure for identifying nutritionally “at risk” women [17, 18]. Cut-offs for MUAC aligned with available government guidance to screen undernourished women that is <23 cm for any form of malnutrition and <19 cm for severe malnutrition [19]. The indicators on access to maternal health services included antenatal and delivery care in alignment with national health surveys. The continuous Food Insecurity Experience Scale (FIES) was used to estimate level of household food insecurity from no food insecurity to severe food insecurity [20].

Modifications were made to the baseline questionnaire after first round of data collection done in Bihar. Most questions and probes on women’s empowerment were added after this and include questions numbered 211 to 226 in the interview schedule for mothers of children under two (S2 Appendix). Thus, limited information is available on the 21 women’s empowerment indicators for Bihar.

### Data management and analysis

Data was entered using Census and Survey Processing System (CS Pro) and analyzed using STATA 15.1. Univariate analysis was used to profile mothers of child under-two by different socio-economic and demographic characteristics. Bivariate analysis was used to show the distribution of SHG and non-SHG member mothers by different indicators of women empowerment, nutrition status, access to maternal health services and household food insecurity. The level of association of women’s empowerment, nutrition status, access to maternal health services and household food insecurity variables with participation of women in SHG was established through Chi square test of independence.

### Ethical considerations

The baseline survey protocol, methodology and tools were approved by the Institutional Ethics Committee of All India Institute of Medical Sciences (AIIMS) in the respective states of Bihar, Chhattisgarh and Odisha. The study has been registered with the Registry for International Development Impact Evaluations (RIDIE-STUDY-ID-58261b2f46876) and Indian Council of Medical Research (ICMR) National Clinical Trials Registry of India (CTRI/2016/11/007482). A consent form detailing the purpose of the study, agency and investigator involved and reasons for anthropometric measurements was developed as part of the study protocol approved by the Ethics Committee. Verbal consent was taken from all interviewees before conducting the interview and taking anthropometric measurements as most respondents were unable to write or sign. Investigators proceeded with the interview only when consent was given by the interviewee.

## Results

### Background characteristics

In all states, majority of mothers of children under-two were in the 20–29 years age group. Odisha had the highest propotion of adolescent mothers (8.9%) among the three states. The proportion of mothers married before legal age was high in both Chhattisgarh and Odisha at 26.4% and 35.7%, respectively. Bihar had the highest proportion of mothers with parity of three or more (52.6%) compared with 38.9% in Chhattisgarh and 33.1% in Odisha. Of every 10 mothers, six in Bihar, five in Odisha and four in Chhattisgarh had never attended school. Mothers mostly belonged to Hindu households in Chhattisgarh and Odisha, and Muslim in Bihar. Over half of the sampled mothers belonged to Scheduled Tribes (ST) in Chhattisgarh and Odisha and Other Backward Castes (OBC) in Bihar. The penetration of PDS was lowest in Bihar with nearly half the mothers not having a ration card. Chhattisgarh had the highest proportion of Antyodaya card holders (17%) or those certified by the goverenment as poorest of the poor households. Nearly one in three mothers were members of SHGs in all states; VO membership was strongest in Bihar with nearly half the SHG members participating in VOs (Table 4).

**Table 4:** Profile of mothers of children under-two by selected background characteristics in Bihar, Chhattisgarh and Odisha.

Background characteristics	Bihar	Chhattisgarh	Odisha
	N (%)	N (%)	N (%)
**Age group (in years)**			
15–19	55 (2.1)	129 (5.1)	321 (8.9)
20–29	1656 (63.4)	1904 (75.0)	2552 (70.8)
30 & above	901(34.5)	505 (19.9)	732 (20.3)
**Age at marriage**			
Below 18 years	NA	670 (26.4)	1287 (35.7)
**Educational status**			
Never attended school	1575 (60.3)	1005 (39.6)	1914 (53.1)
**Religion of the head of household**			
Hindu	1042 (39.9)	2478 (97.6)	3431 (95.2)
Muslim	1570 (60.1)	8 (0.3)	11 (0.3)
Christian	NA	46 (1.8)	151 (4.2)
Others	NA	8 (0.3)	11 (0.3)
**Ethnicity of the head of the household**			
Scheduled caste	502 (19.2)	779 (30.7)	728 (20.2)
Scheduled tribe	128 (4.9)	1650 (65.0)	1914 (53.1)
Other backward classes	1740 (66.6)	0	649 (18.0)
General	243 (9.3)	0	314 (8.7)
**Ration card holder**			
No card	1233 (47.2)	310 (12.2)	580 (16.1)
Antyodaya card	60 (2.3)	421 (16.6)	299 (8.3)
**Parity**			
1	603 (23.1)	853 (33.6)	1315 (36.5)
2	635 (24.3)	701 (27.6)	1096 (30.4)
≥3	1374 (52.6)	988 (38.9)	1193 (33.1)
**SHG, VO membership**			
Member of SHGs	789 (30.2)	706 (27.8)	1081 (30.0)
Member of VOs	384 (48.7)	278 (39.4)	200 (18.5)
**Total (N)**	**2612**	**2539**	**3604**

### Women’s empowerment

#### Women’s agency

Over 70% of mothers irrespective of SHG membership status were involved in household decision making in Odisha. Participation was near or over 70% in Chhattisgarh too, but lower in Bihar, particularly for decision making on social visits to family members and relatives (50% among SHG members and 44% among non-SHG members). When decision making was compared between SHG members and non-SHG members, in Bihar SHG members had greater involvement in decision making on major purchases, their own health care as well as planning social visits. In Chhattisgarh and Odisha, a higher proportion of SHG members could decide on use of money earned by husband than non-SHG members; however, there was no difference in decision making related to major purchases for the house between members and non-SHG members. In Chhattisgarh, like in Bihar, higher proportion of SHG members decided on visits to family members and relatives than non-members (Table 5).

**Table 5:** Empowerment among SHG and non-SHG member mothers of children under-two in Bihar, Chhattisgarh and Odisha.

		Bihar		Chhattisgarh		Odisha	
		Non SHG	SHG	Non SHG	SHG	Non SHG	SHG
		N (%)	N (%)	N (%)	N (%)	N (%)	N (%)
**Agency**	**Participation in household decision making**						
	Decision about using money husband earns	NA	NA	1253 (68.3)	520 (73.8)***	1897 (75.2)	846 (78.3)*
	Decision about making major purchases for the households	1220 (66.9)	556 (70.6)*	1341 (73.1)	538 (76.3)	1910 (75.7)	836 (77.3)
	Decision about own health care	1122 (61.5)	517 (65.6)**	1234 (67.3)	498 (70.6)	1832 (72.6)	792 (73.3)
	Decision about visit to family members and relatives	804 (44.1)	392 (49.8)***	1300 (70.9)	542 (76.9)***	2001 (79.3)	864 (79.9)
	**Autonomy**						
	Can go to market alone or with someone	NA	NA	1515 (82.6)	624 (88.5)***	2268 (89.9)	1011 (93.5)***
	Can go to health facility alone or with someone	NA	NA	1816 (99.0)	700 (99.3)	2450 (97.1)	1049 (97.0)***
	Can go outside alone or with someone	NA	NA	1810 (98.7)	693 (98.3)	2359 (93.5)	1023 (94.6)***
	**Acceptability of domestic violence**						
	Goes out without telling him	NA	NA	323 (17.6)	98 (13.9)**	1153 (45.7)	542 (50.1)**
	Neglects the house or the children	NA	NA	374 (20.4)	125 (17.7)	1284 (50.9)	563 (52.1)
	Argues with him	NA	NA	319 (17.4)	98 (13.9)**	1214 (48.1)	547 (50.6)
	Refuses to have sex with him	NA	NA	216 (11.8)	54 (7.7)***	732 (29.0)	316 (29.2)
	Doesn't cook properly	NA	NA	257 (14.0)	72 (10.2)**	780 (30.9)	349 (32.3)
	Husband suspects her of being unfaithful	NA	NA	314 (17.1)	102 (14.5)	833 (33.0)	365 (33.8)
	Disrespect for in-laws	NA	NA	363 (19.8)	120 (17.0)	1463 (58.0)	650 (60.1)
**Income**	Has own money which she can decide how to spend	NA	NA	906 (49.4)	401 (56.9)***	1297 (51.4)	630 (58.3)***
	Has savings bank account	NA	NA	884 (48.2)	440 (62.4)***	1923 (76.2)	929 (85.9)***
**Work status**	Engaged in economically productive activities in last 12 months	NA	NA	761 (41.5)	310 (44.0)	633 (25.1)	337 (31.2)***
**Leadership**	Organized or participated in organization of any community level program	NA	NA	754 (41.1)	395 (56.0)***	401 (15.9)	306 (28.3)***
**Access to resources**	Has a mobile phone which she alone can use	NA	NA	240 (13.1)	85 (12.1)	547 (21.7)	253 (23.4)
	Owns house (alone/jointly)	NA	NA	867 (47.3)	320 (45.4)	1650 (65.4)	694 (64.2)
	Owns land (alone /jointly)	NA	NA	779 (42.5)	271 (38.4)*	1171 (46.4)	503 (46.5)
**Total (N)**		**1824**	**788**	**1834**	**705**	**2523**	**1081**

Note

* p<0.10

** p<0.05; and

*** p<0.01 level of significance

Mothers did well on autonomy with nearly all being able to visit a health facility or going outside the house either alone or with someone in both Chhattisgarh and Odisha. In Odisha, a higher proportion of SHG members were able to undertake these tasks than non-SHG members. In both Chhattisgarh and Odisha, SHG members reported being able to go to market alone or with someone more frequently than non-SHG members.

Endorsement for domestic violence was higher among mothers in Odisha than Chhattisgarh. Among the seven situations presented, in Odisha, mothers endorsed domestic violence most commonly for disrespecting in-laws (58% and 60%), followed by neglecting house or children (51% and 52%), arguing with husband (48% and 51%) and leaving house without informing husband (46% and 50%). They were least likely to endorse it for refusal of sex which was still relatively high at 29%. The situations endorsed by mothers in Chhattisgarh were similar but proportion mothers endorsing domestic violence were much lower and less than 20% across situations. Further, the endorsement for domestic violence lowered among mothers who were SHG members for four of the seven situations in Chhattisgarh, unlike one of seven situations in Odisha (Table 5).

#### Income

In Chhattisgarh and Odisha, half the mothers had own income which they could independently spend and higher proportion of SHG members did so opposed to non-SHG members. Higher proportion of SHG members had a savings bank account than non-SHG members, 62% among SHG members opposed to 48% among non-SHG members in Chhattisgarh and 86% among SHG members opposed to 76% among non-SHG members in Odisha (Table 5).

#### Work status

More women in Chhattisgarh than Odisha were engaged in economically productive work in the 12 months preceding the survey. The proportion engaged in such work was similar among SHG members and non-SHG members in Chhattisgarh (44% and 42%, respectively). However, higher proportion of SHG members were working in Odisha than non-SHG members (31% opposed to 25%) (Table 5).

#### Leadership

A higher proportion of mothers organized or participated in community mobilization activities in Chhattisgarh than in Odisha. In both states, higher proportion of SHG members were involved in such activities than non-SHG members; 56% in Chhattisgarh and 28% in Odisha (Table 5).

#### Access to resources

Ownership of resources that is mobile phone, house and land was overall higher in Odisha than Chhattisgarh but comparable between SHG members and non-SHG members. The exception was Chhattisgarh where higher proportion of SHG members owned land than non-SHG members (38% opposed to 43%). Among investigated resources, ownership of mobile phone was lowest with about 12% and just over 20% owning mobile phones in Chhattisgarh and Odisha, respectively (Table 5).

### Nutritional status

Over half the mothers in Chhattisgarh were thin with BMI < 18.5 kg/m^2^ (55% SHG members and 54% non-SHG members). Prevalence of thinness was also high in Bihar and Chhattisgarh with 45% and 55% mothers having BMI < 18.5 kg/m^2^. Using MUAC, prevalence of thinness was highest in Bihar (57% among SHG members and 61% among non-SHG members). Severe thinness too was considerably higher in Bihar compared with other two states (9% among SHG members and 8% among non-SHG members) (Table 6).

**Table 6:** Nutritional status of SHG and non-SHG member mothers of children under two years in Bihar, Chhattisgarh and Odisha.

	Bihar		Chhattisgarh		Odisha	
	Non SHG	SHG	Non SHG	SHG	Non SHG	SHG
	N (%)	N (%)	N (%)	N (%)	N (%)	N (%)
BMI < 18.5 kg/m^2^ (thin)	808 (44.8)	352 (44.9)	992 (54.2)	389 (55.2)	1131 (45.3)	489 (45.6)
MUAC < 23 cm (thin)	1094 (60.7)	446 (57.0)*	803 (43.9)	283 (40.1)	1006 (40.3)	440 (41.0)
MUAC <19 cm (severely thin)	150 (8.3)	69 (8.8)	15 (0.8)	9 (1.3)	25 (1.0)	14 (1.3)
**Total (N)** ^a^	**1803**	**783**	**1830**	**705**	**2497**	**1073**

Note

* p<0.10

** p<0.05; and

*** p<0.01 level of significance

^a^Sample size was smaller for ascertainment of nutrition status due to participants’ refusal to anthropometry

### Household food security

Odisha had the least proportion of food secure households (13% SHG members households and 17% non-SHG members households) followed by Bihar and Chhattisgarh. Members of SHG more frequently belonged to food insecure households across all states. Among food insecure households, over 70% were moderate to severely insecure in Odisha, over 60% in Bihar and about 40% in Chhattisgarh. While state variations were observed, there was no difference between levels of food insecurity with respect to SHG membership status (Table 7).

**Table 7:** Household food security among SHG and non-SHG member mothers of children under two years in Bihar, Chhattisgarh and Odisha.

	Bihar		Chhattisgarh		Odisha	
	Non SHG	SHG	Non SHG	SHG	Non SHG	SHG
	N (%)	N (%)	N (%)	N (%)	N (%)	N (%)
Food secure household	434 (24.0)	132 (16.8)***	627 (34.3)	163 (23.1)***	431 (17.1)	142 (13.0)***
Mildly food insecure household	294 (16.1)	145 (18.4)	603 (32.9)	255 (36.2)	280 (11.1)	116 (10.7)
Moderately food insecure household	868 (47.6)	423 (53.7)	477 (26.0)	240 (34.0)	1135 (45.0)	480 (44.4)
Severely food insecure household	228 (12.5)	88 (11.2)	127 (6.9)	47 (6.7)	676 (26.8)	344 (31.8)
**Total (N)**	**1824**	**788**	**1834**	**705**	**2523**	**1081**

Note

* p<0.10

** p<0.05; and

*** p<0.01 level of significance

### Maternal health services

Almost all pregnancies were registered in Chhattisgarh and Odisha, but over a quarter of mothers were not brought into the health system during their pregnancy in Bihar. Higher proportion of SHG members were registered than non-SHG members in all states. Less than a quarter of the mothers were registered in the first trimester in Odisha, about a third in Chhattisgarh and Bihar. SHG membership had no influence on the chance of receiving ANC in the first trimester. Weight measurement was done for over 85% of the mothers in Chhattisgarh and Odisha but less frequently in Bihar, with 75% SHG members and 70% non-SHG members reporting weight measurement. Less than one in five mothers underwent height measurement in Chhattisgarh and Odisha; it was rarely measured in Bihar (<3%). Both Blood Pressure (BP) measurement and hemoglobin estimation was reported by at least 80% of the mothers in both Chhattisgarh and Odisha. In Bihar, coverage of these services was lower with a little over 40% mothers reporting BP measurement and hemoglobin estimation. Not much difference was observed in reach of these services with respect to SHG membership status. Tetanus Toxoid (TT) vaccination was almost universal with SHG members covered better than non-SHG members in Bihar and Chhattisgarh. Receipt of IFA varied much across states being lowest in Bihar (53% non-SHG members and 61% SHG members), followed by Chhattisgarh (79% non-SHG members and 86% SHG members) and highest in Odisha (91% non-SHG members and 92% SHG members).

Use of health facilities for delivery was lowest in Chhattisgarh (65% non-SHG members and 62% SHG members). It was relatively higher in Bihar and higher proportion of SHG members used health facilities (85%) than non-SHG members (75%). Use of modern family planning methods was excruciatingly low (10%) in Chhattisgarh and Bihar; in Bihar higher proportion of SHG members were using modern family methods (13%) compared with non-SHG members (8%).

Village Health Sanitation and Nutrition Days (VHSNDs) were attended by 60% or more mothers in Chhattisgarh and Bihar with higher proportion of SHG members participating in both states. Participation was extremely poor in Bihar with less than 10% of mothers attending VHSNDs and no difference with respect to SHG membership (Table 8).

**Table 8:** Access to maternal health services among SHG and non-SHG member mothers of children under two years in Bihar, Chhattisgarh and Odisha.

	Bihar		Chhattisgarh		Odisha	
	Non SHG	SHG	Non SHG	SHG	Non SHG	SHG
	N (%)	N (%)	N (%)	N (%)	N (%)	N (%)
**Pregnancy registered**	1299 (71.2)	590 (74.9)*	1770 (96.5)	694 (98.4)***	2425 (96.1)	1057 (97.8)**
**ANC services**						
ANC check-up in 1^st^ trimester	482 (37.1)	239 (40.5)	557 (31.5)	219 (31.6)	567 (23.4)	233 (22.0)
Weight measured	904 (69.6)	440 (74.6)**	1541 (87.1)	645 (92.9)***	2051 (84.6)	915 (86.6)
Height measured	32 (2.5)	17 (2.9)	364 (20.6)	144 (20.8)	453 (18.7)	200 (18.9)
BP measured	546 (42.0)	262 (44.4)	1527 (86.3)	587 (84.6)	1927 (79.5)	853 (80.7)
Hemoglobin tested	549 (42.3)	229 (38.8)	1509 (85.3)	577 (83.1)	2036 (84.0)	898 (85.0)
TT injection received	1285 (98.9)	576 (97.6)**	1728 (97.7)	688 (99.1)**	2276 (93.9)	999 (94.5)
IFA received	692 (53.3)	358 (60.7)***	1399 (79.1)	599 (86.3)***	2201 (90.8)	969 (91.7)
**Total (N)**^**a**^	**1299**	**590**	**1769**	**694**	**2424**	**1057**
**Institutional delivery**	1363 (74.7)	667 (84.6)***	1201 (65.5)	440 (62.4)	1809 (71.7)	756 (69.9)
**Current use of modern family planning**	150 (8.2)	101 (12.8)***	178 (9.7)	69 (9.8)	691 (27.4)	350 (32.4)***
**Attended VHSND Meetings**	115 (6.3)	62 (7.9)	1135 (61.9)	462 (65.5)*	1415 (56.1)	689 (63.7)***
**Total (N)**	**1824**	**788**	**1834**	**705**	**2523**	**1081**

Note

* p<0.10

** p<0.05; and

*** p<0.01 level of significance.

^a^ Sample size was smaller as estimates were drawn from registered pregnancies.

## Discussion

We found that at least one in four mothers were members of SHGs in Bihar, Chhattisgarh and Odisha; there may be more who came from households that had an SHG member as NRLM aims to have at least one woman member from each family linked to the SHGs. Our survey did not capture the latter. However, the context in which Swabhimaan operates is marked by social and gender inequalities. In all Swabhimaan intervention states, that is Bihar, Chhattisgarh and Odisha, sampled population belonged to government enlisted vulnerable communities of SC, ST or OBC. Over a third of mothers in Odisha were married before legal age and nearly one in ten were adolescent mothers. Schooling rates were low for mothers in all three states, one of the known drivers for early marriages and childbearing [21].

In our survey, 11 of the 21 indicators for women’s empowerment in Chhattisgarh and 9 of the 21 in Odisha were skewed towards mothers who were SHG members while no difference was reported in the others. Estimates were available for only three of the 21 indicators in Bihar, all related to decision making and positively skewed towards SHG members. Indicators which consistently demonstrated better women’s empowerment status among SHG members in both Chhattisgarh and Odisha were: 1) Having a bank account, 2) Having own money and control of this money and 3) Organizing or participating in community level programs as a proxy for community leadership. Among women’s agency indicators a relatively high proportion of mothers reported autonomy as well decision making authority, however, acceptance of domestic violence was quite high especially in Odisha and it did not vary much with SHG membership. In our earlier work we had noted that guarding women from domestic violence is critical for successful implementation of women’s centered livelihood programs [5]. The JEEViKA assessment also indicated that the program was successful in mobilizing marginalized women into institutional platforms, such women demonstrated higher levels of empowerment, when empowerment was measured by mobility, decision making and collective action [12]. Access to mobile phones was low in both Chhattisgarh and Odisha thus, limiting use of this technology for the initiative. SHG members were less likely to own land in Chhattisgarh, indicating that the program is reaching to more vulnerable households first. This was also supported by the findings on higher proportion of SHG member mothers belonging to food insecure households than non-SHG members across all states. Alternately, the longevity of membership was either not sufficient to influence food security or membership did not impact food security. This needs to be further investigated. Evaluations of poverty reduction programs revealed varying results with no change in food consumption for poor households in China to improved food and nutrient intake for SHG members and reduced food shortages in India [7, 13, 22]. The coverage of National Food Security Act (NFSA), 2013 as measured by availability of ration card for PDS varied across states with nearly half the population in Bihar unreached. Under the NFSA highly subsidized food grains are provided to food insecure households on priority while it aims to cover 75% of rural and 50% of urban population[23].

We observed high prevalence of undernutrition among mothers, scathing at 45% or higher across states. Using MUAC the situation worsened for Bihar with six in 10 women thin and one in ten severely thin. Unsurprisingly, not much variation was seen in nutrition status of SHG members and non-SHG members as there was no nutrition centered intervention in these areas except the universally applicable health and welfare schemes. Among the antenatal services, across all states, TT coverage was the highest and almost universal while height was most infrequently measured. Height <145 cm is an independent risk factor for low birth weight of babies and proposed to be used by the national government for screening nutritionally “at risk” women [24, 25, 26]. Height measurement is also essential for calculating BMI before or in early pregnancy to estimate optimal gestation weight gain [17]. More mothers delivered in an institution than were brought into the health system during delivery in Bihar; further higher proportion of SHG members delivered in a health facility. This may be partly explained by the financial incentives linked to institutional delivery under Janani Suraksha Yojana (a maternity benefit, cash incentive scheme) [27]. However, the situation in Chhattisgarh and Odisha was contradictory with mothers registered during pregnancy but not availing facility health services for delivery. The use of modern family planning methods among mothers was low especially in Bihar which explains the high parity in the sampled population. VHSND which is the most accessible platform for mothers to gain information and receive basic services on health, nutrition and family planning was excruciatingly low in Bihar. Improving quality and coverage of VHSND is one of the interventions under Swabhimaan. The Swabhimaan interventions are also aligned with the Government of India’s newly launched multi-sectoral nutrition initiative–Poshan Abhiyan which envisions NRLM’s role in strengthening VHSNDs, integrated behavior promotion strategies across NRLM, converging with Health and Women and Child Development Departments and promoting nutri-sensitive livelihoods [28].

## Conclusion

NRLM’s poverty reduction program has some gains in improving women’s empowerment, particularly on increasing women’s control over financial resources and community leadership. Women’s tolerance to domestic violence in Odisha was a concern and needs to be addressed through NRLM or other social welfare platforms. Along with increasing SHG membership, need for improving reach of food security schemes or nutri-sensitive livelihoods is needed across all states. The gains in women’s financial control are yet to translate in improving their nutritional status across all states. SHG membership has varying influence in use of antenatal, partum and family planning services across Bihar, Chhattisgarh and Odisha. Services that require increased attention across all states include pregnancy registration in first trimester, height measurement of pregnant women, increased information and access to family planning methods and participation in VHSNDs. In Bihar, reach of all ANC services excluding TT vaccination need to be reviewed. The need for layering essential women’s nutrition interventions that is access to health services and care to nutritionally “at risk”, access to family planning services, improving dietary diversity, preventing micronutrient deficiencies and anemia and access to water, sanitation and hygiene services is immense. The context supports an inititaivet like Swabhimaan to layer these nutrition interventions on the NRLM platforms. This may be more effective in a state like Bihar where nearly half the SHGs have federated to VOs.

## Supporting information

S1 Appendix Swabhimaan baseline survey, 2016–17, Chhattisgarh. Household questionnaire.
(PDF)

S2 Appendix Swabhimaan baseline survey, 2016–17, Chhattisgarh. Mothers of under two years questionnaire.
(PDF)

S3 Appendix Swabhimaan baseline survey data tables 2016–17 (Bihar, Chhattisgarh, Odisha). (RAR)

## References

[1] International Institute for Population Sciences and Macro International. National Family Health Survey 4, 2015–16. India Fact sheet Mumbai; 2017.

[2] Ministry of Health and Family Welfare. Health and family welfare statistics of India 2015 New Delhi: Ministry of Health and Family Welfare, Statistics Division, 2015

[3] Inter-Parliamentary Union and World Health Organization. Child, early and forced marriage legislation in 37 Asia-Pacific countries: World Health Organization Geneva; 2016.

[4] Butta Z, Das JK, Rizvi A, Gaffey MF, Walker N, Horton S, et al Evidence-based interventions for improvement of maternal and child nutrition: what can be done and at what cost? Lancet. 2013; 382: 452–477. 10.1016/S0140-6736(13)60996-4 23746776

[5] Sethi V, Bhanot A, Bhalla S, Bhattacharjee S, Daniel A, Sharma DM, et al Partnering with women collectives for delivering essential women’s nutrition interventions in tribal areas of eastern India: a scoping study. J Health Popul Nutr. 2017; 36 (20). 10.1186/s41043-017-0099-8 PMC5441055 28532433

[6] George V. Kudumbashree: How rethinking poverty and gender changed 5 million lives in Kerala. The Better India. 2017 Available from: https://www.thebetterindia.com/119677/kudumbashree-poverty-gender-5-million-kerala/.

[7] Deininger K, Liu Y. Economic and social impacts of an innovative self-help group model in India. World Development. 2013; 43: 149–163.

[8] National Rural Livelihoods Mission, Ministry of Rural Development, Government of India. Briefing book. 2012

[9] National Rural Livelihoods Mission. [accessed 06.08.2018]. Available from: https://nrlm.gov.in/outerReportAction.do?methodName=showIndex.

[10] Swabhimaan (2016 to 2020) unpublished report by UNICEF. India 2017.

[11] Kumar N, Scott S, Menon P, Kannan S, Cunningham K, Tyagi P, et al Pathways from women's group-based programs to nutrition change in South Asia: A conceptual framework and literature review. Global Food Security. 2017 Available from: 10.1016/j.gfs.2017.11.002. PMC6004534 29930896

[12] Datta U. Socio-economic effects of a Self-help group intervention: Evidence from Bihar, India The World Bank.

[13] Kishor S and Gupta K. Women's Empowerment in India and Its States: Evidence from the NFHS. Economic & Political Weekly. 2004; 39 (7): 694–712.

[14] Integrated multisectoral strategy to improve girls’ and women’s nutrition before conception, during pregnancy and after birth in India (Swabhimaan): prospective, non-randomised controlled evaluation. Principal investigator: Sethi V. Co-principal investigator: Unisa S. Available from: http://ridie.3ieimpact.org/index.php?r=search/detailView&id=485 10.1136/bmjopen-2019-031632 PMC6886981 31740469

[15] World Health Organization (WHO). Physical status: the use and interpretation of anthropometry Report of a WHO Expert Committee. World Health Organ Technical Report Series. Geneva: WHO, 1995. 8594834

[16] Institute of Medicine. Weight Gain During Pregnancy. Re-examining the guidelines Washington, DC: National Academies Press; 2009:370.

[17] Kumar P, Sareen N, Agrawal S, Kathuria N, Yadav S, Sethi V. Screening maternal acute malnutrition using adult mid-upper arm circumference in resource-poor settings. Indian J Community Med. 2018; 43:132–134. 10.4103/ijcm.IJCM_248_17 29899619 PMC5974833

[18] Tang AM, Chung M, Dong K, Terrin N, Edmonds A, Assefa N, et al Determining a Global Mid Upper Arm Circumference Cut Off to Assess Malnutrition in Pregnant Women Washington, DC: FHI 360/Food and Nutrition Technical Assistance III Project (FANTA); 2016.

[19] Ministry of Health and Family Welfare. Government of India. Guidance document: Nutritional care and support for patients with tuberculosis in India World Health Organization; 2017.

[20] Food and Agriculture Organization. The Food Insecurity Experience Scale: Measuring food insecurity through people’s experience; 2017.

[21] Wodon QT, Male C, Nayihouba KA, Onagoruwa AO, Savadogo A; Yedan A, et al Economic impacts of child marriage: global synthesis report (English) 2017 Economic Impacts of Child Marriage Washington, D.C: World Bank Group [accessed 06.08.2018]. Available from: http://documents.worldbank.org/curated/en/530891498511398503/Economic-impacts-of-child-marriage-global-synthesis-report.

[22] Park A and Wang S. Community-Based Development and Poverty Alleviation: an Evaluation of China's Poor Village Investment Program (June 2010). CEPR Discussion Paper No. DP7856. [accessed 06.08.2018]. Available from: https://ssrn.com/abstract=1640378.

[23] About NFSA. [accessed 06.08.2018]. Available from: http://dfpd.nic.in/nfsa-act.htm.

[24] Kamaladoss T, Abel R, Sampath VK. Epidemiological co-relates of low birth weight in rural Tamil Nadu. Indian J Pediatr. 1992; 59:299–304. 1398861 10.1007/BF02821792

[25] World Health Organization (WHO). Maternal anthropometry and pregnancy outcome: a WHO collaborative study. Bull World Health Organ. 1995;73(Suppl):1–98. PMC2486648 8529277

[26] Maternal Nutritional Care at NRC. National Nutritional Rehabilitation Resource & Training Centre, Kalawati Saran Children’s Hospital in partnership with UNICEF and Lady Irwin College, New Delhi; 2018.

[27] About JSY. Available from: http://ayushmanbharat.net/wp-content/uploads/2018/02/JSR.pdf.

[28] About Poshan Abhiyan. Available from: https://www.icds-wcd.nic.in/nnm/NNM-Web-Contents/LEFT-MENU/Review-Meetings/EC_30-05-2018/POSHAN_Abhiyaan-JanAndolanGuidelines.pdf.

