# Supplementary material for: Context for layering women’s nutrition interventions on a large scale poverty alleviation program: Evidence from three eastern Indian states
Source: PLoS One. 2019 Jan 22;14(1):e0210836. doi: 10.1371/journal.pone.0210836 (PMC6342298; doi:10.1371/journal.pone.0210836)
Supplement: S1 Appendix — Household questionnaire. (PDF) [file pone.0210836.s001.pdf]

CONFIDENTIAL FOR RESEARCH PURPOSE
शोधकार्य हेतु गोपनीय

SWABHIMAAN, BASE LINE SURVEY, 2016-17, Chhattisgarh
HOUSEHOLD QUESTIONNAIRE

स्वाभिमान, बेसलाइन सर्वेक्षण, 2016–17, छत्तीसगढ़

परिवार प्रश्नावली

A. IDENTIFICATION पहचान

1. STATE राज्य..
2. DISTRICT जिला..
3. BLOCK ब्लॉक..

3.1 AREA (1 = INTERVENTION 2 = CONTROL) क्षेत्र (1= हस्तक्षेप, 2=कंट्रोल)..

4. PANCHAYAT पंचायत..
5. VILLAGE NAME & CODE गाँव का नाम एवं कोड____
6. LOCATION OF THE HOUSE (1= MAIN VILLAGE 2= HAMLET) मकान की स्थिति (1=मुख्य गाँव, 2=टोला/पारा)..

6.1 NAME OF HAMLET (IF HOUSE LOCATED IN HAMLET OF VILLAGE) टोले/ पारे का नाम (यदि घर गाँव के टोले में स्थित है तो) ____

7. HOUSE NUMBER मकान संख्या ....
8. NAME OF THE INVESTIGATOR साक्षात्कारकर्त्ता का नाम ______________________________
9. MOBILE NUMBER AVAILABLE मोबाईल नंबर उपलब्ध है (YES हॉ = 1 NO नहीं=0)..

9.1 MOBILE NUMBER 1 मोबाईल नंबर 1 ____________________

MOBILE NUMBER 2 मोबाईल नंबर 2 ____________________

10. LANDMARK TO LOCATE THE HOUSEHOLD मकान के पास का लैंडमार्क ______________________

10.1 NAME OF THE HEAD OF HOUSEHOLD घर के मुखिया का नाम ______________________________

11. RESPONDENT उत्तरदाता (1= HOUSEHOLD HEAD घर का मुखिया 2 = OTHER MEMBER OF HH घर के अन्य)..........

11.1 NAME OF THE RESPONDENT उत्तरदाता का नाम ______________________________

12. OWNERSHIP OF THE DWELLING UNIT आवासीय ईकाई का मालिक [1= SELF OWNED खुद ही मालिक हैं 2=RENTED किराये पर 3= OTHER अन्य]..
13. DURATION OF STAY IN THE VILLAGE गाँव में रहने का समयाअवधि (IF DURATION OF STAYING IS LESS THAN ONE YEAR ENTER '000') YYY[वर्ष]..
14. HOW MANY PEOPLE LIVE IN YOUR HOUSEHOLD (INCLUDING INFANTS AND YOURSELF) आपके घर में आपको और नवजात शशुओं को मिलाकर कुल कितने लोग रहते हैं?..

14.1 SERIAL NUMBER OF QUESTIONNAIRE प्रश्नावली क्रमांक ....

B. RESULT STATUS

पत्रक की परिणाम स्थिति

**R1.**	**HOUSEHOLD QUESTIONNAIRE** घर की प्रश्नावली	COMPLETED पूर्ण	1
		PARTLY COMPLETED आंशिक पूर्ण	2

	KINDLY CHECK THE BOX AGAINST SELECTED TARGET GROUP FROM THE HOUSEHOLD कृपया परिवार से चयनित लक्ष्य समूह से संबंधित बॉक्स का अवलोकन करे		A. RESULT STATUS परिणाम की स्थिति AVAILABLE उपलब्ध = 1 TEMPORARILY AWAY अस्थायी तौर पर बाहर = 2 MIGRATED OUT पलायन किया = 3	B. NAME OF THE WOMAN महिला का नाम	C. LINE NUMBER OF WOMAN (Serial number in Q15 of HH Questioner) महिला की प्रश्नावली में क्रम संख्या (परिवार प्रश्नावली के प्रश्न क. 15 में क्रम संख्या)
**R2.**	ADOLESCENT किशोरी	☐	☐	______________________________	☐☐
**R3.**	PREGNANT WOMAN गर्भवती महिला	☐	☐	______________________________	☐☐
**R4.**	MOTHER OF CHILD UNDER 2 YEARS 2 वर्ष के बच्चे की माता	☐	☐	______________________________	☐☐

R5. INTERVIEW DATE साक्षात्कार दिनॉक

D	D	M	M	Y	Y	Y	Y

R6. NUMBER OF VISITS MADE भ्रमणों की संख्या ☐☐

R7. SPOT CHECKED BY (IIPS) स्पाट चैक (IIPS)______________________

R9. FIELD EDITED BY फिल्ड एडिट किसके द्वारा______________________

R8. BACK CHECKED BY (IIPS) बैक चैक (IIPS)______________________

R10. OFFICE EDITED BY आफिस एडिट किसके द्वारा______________________

R11. CODE OF INVESTIGATOR साक्षात्कारकर्ता का कोड ☐☐

INTRODUCTION AND INFORMED CONSENT
[परिचय एवं सूचित सहमति]

Namaskar! My name is ___________________________ and I am working with All India Institute of Medical Sciences (AIIMS) in the ***"Swabhimaan"*** Project, funded by UNICEF. We are conducting a study/survey about the health & nutritional status of Adolescent girls (10-19) years, Pregnant Women & Lactating Mothers (mother of child under two years) in the Bastar district of Chhattisgarh state.

For Swabhimaan Project we would like to ask you some questions about your household members. We would also like to interact with Adolescent Girls (10-19 years), Pregnant Women & Lactating Mothers (mother of child under two years) in your household.

The information shared by you will remain confidential and would be used only for programme, planning and research purposes. Any personal identifiers that could reveal your identity would be removed before the results of the study are made public or shared between people other than the main researchers working on the project.Your participation is entirely voluntary and you can choose to discontinue your participation at any time without giving a reason. This interview will take around 20-25 minutes to complete. If you have any questions about the survey feel free to ask me.

We thank you for taking time to understand and showing your interest in the study.

(परिचय) **''नमस्कार/राम राम/जोहार''** मेरा नाम ___________________________ है । मैं **''स्वाभिमान परियोजना'' में एम्स रायपुर** के साथ काम कर रहा/रही हूँ । यह स्वाभिमान परियोजना बस्तर जिले में यूनिसेफ और बिहान के द्वारा चलाई जा रही है। इस परियोजना में हम लोग 10 –19 वर्ष की किशोरियों, गर्भवती महिलाओं और 2 वर्ष की उम्र के बच्चों की माताओं के स्वास्थ्य और पोषण की स्थिति अध्ययन करने के लिए सर्वेक्षण कर रहे हैं।

(उद्देश्य) आज हम स्वाभिमान परियोजना के लिए आपसे आपके घर के सदस्यों के बारे में कुछ जानकारियों के लिए आपका साक्षात्कार करेंगे। और हम आपके घर की चयनित किशोरियों, गर्भवती महिलाओं और 2 वर्ष की उम्र के बच्चों की माताओं से भी साक्षात्कार करगे।

(गोपनीयता) साक्षात्कार में आपके द्वारा प्रश्नों पर दी गइ जानकारियों को गोपनीय रखा जाएगा। इन जानकारियों का उपयाग केवल स्वास्थ्य और पोषण संबंधी कार्यक्रमों, योजनाओं और अध्ययन के लिए किया जाएगा।

आपकी पहचान कभी भी किसी भी स्थिति में प्रकट नही की जाएगी। ऐसी सभी व्यक्तिगत जानकारियां जो आपको इस अध्ययन के प्रतिभागी के रूप में पहचानने में मदद कर सकती ह, उन्हे किसी अन्य व्यक्ति से साझा करने अथवा प्रकाशित करने से पहले हटा दिए जाएगा।

(पूर्णतः स्वैच्छिक सहभागिता)

इस सर्वेक्षण में आपकी सहभागिता पूर्णतः स्वैच्छिक और आपकी ईच्छा पर आधारित है। इस साक्षात्कार को पूरा करने में 20 से 25 मिनट लगेंगे, जिसमें भाग लेने या ना लेने का निर्णय आप पर है। आपकी भागेदारी स्वैच्छिक है और आप किसी भी समय बिना किसी कारण बताए साक्षात्कार में अपनी सहभागिता के लिए मना कर सकती है।

(उत्तरदाता से सहमति)

यदि आप सर्वेक्षण या साक्षात्कार के बारे में कोइ प्रश्न पूछना चाहे तो अवश्य पूछे। और अगर आप सहमत है तो हम आपका साक्षात्कार प्रारंभ कर सकते है।

हम आपका आभार व्यक्त करते हैं कि आपने इसे समझने में समय दिया और इस अध्ययन में अपनी रूचि दिखाई।

**VERBAL CONSENT TAKEN मौखिक सहमति ली गई**	**CONSENT GIVEN (सहमति दी गई)**	**1**
	**CONSENT NOT GIVEN (सहमति नहीं दी गई)**	**2**

NAME OF THE RESPONDENT उत्तरदाता का नाम :___________________________

SIGNATURE OF THE INVESTIGATOR साक्षात्कारकर्ता के हस्ताक्षर:___________________________

DATE दिनाँक :___________________________

RECORD THE START TIME साक्षात्कार प्रारंभ करने का समय अंतिक करं

(In 24 hour format) (24 घंटे के प्रारूप अनुसार): HOUR घंटा ☐☐ **MINUTES मिनट** ☐☐

Household schedule (respondent may consult other family member(s) of the household to give correct information about each member)

घरेलु सर्वेक्षण प्रपत्र (उत्तरदाता परिवार के सभी सदस्यों के बारे में सही जानकारी देने के लिए घर के अन्य सदस्यों से संपर्क कर सकते हैं)

Now I would like to have some information about the people who usually live in your household अब मैं आपके घर में समान्यता रहनेवाले सदस्यों के बारे में कुछ जानकारी चाहूँगा।

LINE NO.<br>क्रम सं.	Name<br>**Now, please give me the names of all your HH members who live in this household, starting with Head of the Household.**<br>नाम<br>कृपया घर में रहने वाले सभी सदस्यों के नाम बताएं, घर के मुखिया के नाम से प्रारंभ करें। (अगले कालम से एक एक सदस्य के लिए पंक्तिवार प्रश्न 17 से 29 के कालम पूर्ण करे)	DATE OF BIRTH<br>**(verify with the document if possible)**<br>जन्म तिथि (संभव हो तो उपलब्ध दस्तावेज देखकर दर्ज कर)								Age<br>**(in completed years)**<br>**(Record '00' for age less than one year)**<br>उम्र<br>(पूर्ण वर्षों में)– 1 वर्ष से कम आयु के लिए 00 दर्ज करें।	Sex<br>MALE=1<br>FEMALE=2<br>OTHER=3<br>लिंग<br>पुरुष =1<br>महिला=2<br>अन्य =3	Relation Ship with the head of the household<br>**(CODE)**<br>घर के मुखिया से संबंध<br>(कोड)	Marital Status<br>**(For persons aged 10 years and above)**<br>**(CODE)**<br>वैवाहिक स्थिति<br>(10 वर्ष और उससे से अधिक आयु वालों के लिए)<br>(कोड)	**(Only if the child is below 2 yeas)**<br>Name of Mother<br>यदि बच्चा 2 साल से कम उम्र का है तो माता का नाम	**(Only for women aged 10 Years and above)**<br>(Name) Is Currently Pregnant?<br>YES=1<br>NO=0<br>**(महिला जिनकी आयु 10 वर्ष या अधिक हो)**<br>क्या ये (नाम) अभी गर्भवती हैं?<br>1 – हाँ<br>0 – नहीं	**(Only for women aged 10 years and above)**<br>Does [Name] have a child under the age of two?<br>YES=1<br>NO=0<br>**(महिला जिनकी आयु 10 वर्ष या अधिक हो)**<br>क्या ये (नाम) अभी 2 वर्ष से कम उम्र के बच्चे की माता हैं?<br>1 – हाँ<br>0 – नही	Is [Name] a member of a SHG?<br>YES=1<br>NO=0<br>DK=8<br>**(For women aged 10 years and above)**<br>**(IF "NO" SKIP TO 28)**<br>क्या ये (नाम) किसी **स्वयं सहायता समूह** की सदस्या हैं?<br>**(यदि घर की महिला सदस्य जिनकी आयु 10 वर्ष या अधिक हा तो ही ये प्रश्न करे,)**<br>1 – हाँ<br>0 – नही<br>8 – नहीं पता<br>**(यदि नही तो प्र. 28 पर जाएँ)]**	Is [name] a member of a VO/CLF?<br>YES=1<br>NO=0<br>DK=8<br>क्या ये (नाम) किसी ग्राम/क्लस्टर संगठन की सदस्या हैं?<br>1 – हाँ<br>0 – नही<br>8 – नहीं पता	Has (Name) Taken Loan From JEEVIKA<br>YES=1<br>NO=0<br>क्या इन्होने (नाम) बिहान (अजिविका मिशन) से ऋण (लोन) लिया है?<br>1 – हाँ<br>0 – नही	**(IF YES IN Q26)**<br>Type of loan taken?<br>YES=1 NO=0 **(MULTIPLE OPTIONS)**<br>**(यदि प्रश्न 26 में हाँ हो तो)**<br>किस तरह का लोन लिया है?<br>हाँ=1 नहीं=0<br>(कई विकल्प संभव)							Is (Name) Member of Farmer Producer Group?<br>YES=1<br>NO=0<br>**(For persons aged 18 years and above)**<br>क्या ये (नाम) किसी **किसान उत्पादक समूह** क सदस्य हैं?<br>**(सदस्य जिसकी आयु 18 वर्ष या अधिक हो)**<br>1 – हाँ<br>0 – नही	**In the last five years, did [Name] migrate anywhere outside this village for more than a month? (For person aged 5 years and above) YES=1 NO=0**<br>क्या (नाम) नें गत 5 वर्ष में गाँव के बाहर एक माह से अधिक समय के लिए पलायन किया है। (5 वर्ष से अधिक आयु के लिए)<br>1 – हाँ<br>0 – नही	**What was the reason for [Name's] migration ?**<br>(नाम) के पलायन का कारण क्या था?<br>**(CODE)**	Q29 (b) का उत्तर कोड 10 होने पर उल्लेख करे
		D	D	M	M	Y	Y	Y	Y											CIF	VRF	RF	HRF	FSF	HF	BL				
**Q15**	**Q16**	**Q17**								**Q 17.1**	**Q18**	**Q19**	**Q20**	**Q21**	**Q22**	**Q23**	**Q24**	**Q25**	**Q26**	**Q27**							**Q28**	**Q29 (a)**	**Q29 (b)**	**Q29 (c)**

CODE FOR Q19 प्रश्न संख्या 19 के लिए कोड

Item वर्ग	Code	Item	Code
Self स्वय	1	Other relatives<br>अन्य संबंधी	11
Wife or Husband<br>पति या पत्नी	2	Adopted/ foster child<br>गोद लिया गया बच्चा	12
Son or Daughter<br>बेटा या बेटी	3	Not related<br>संबंधी नहीं ह।	13
Son-in-law or Daughter-in-law<br>दामाद या बह	4		
Grandchild<br>पोता-पोती/नाती-नातिन	5		
Parent<br>माता–पिता	6		
Parent-in-law सास–ससुर	7		
Brother or Sister<br>भाई या बहन	8		
Brother-in-law or Sister-in-law<br>साला/जीजा/देवर या साली/ननद/भाभी	9		
Niece or Nephew<br>भतीजा या भतीजी	10		

CODE FOR Q20

Item(वर्ग)	Code
Never married<br>शादी नहीं हुई	1
Married but, gauna not performed<br>शादी हो गई पर गौना नहीं हुआ है	2
Married and gauna performed<br>शादी और गौना, दोनो हुआ है	3
Remarried<br>दुबारा शादी हुई है	4
Widow / widower<br>विधवा/विधुर	5
Divorced तलाकशुदा	6
Separated<br>अलग रह रही/रहा हैं	7
**Live-in relationship**<br>बिना विवाह के साथ साथ रहते हैं।	8
Not Stated<br>नहीं बताया गया	9

FULL FORMS OF CODES OF Q25

VO	Village Organisation<br>ग्राम संगठन
CLF	Cluster Level Federation<br>क्लस्टर लेवल फेडरेशन

FULL FORMS OF CODES OF Q27
प्रश्न संख्या 27 के लिए कोड

Item	Full forms प्रकार
CIF	Community Investment Fund<br>सी. आई. एफ.
VRF	Vulnerability Reduction Fund<br>वी. आर. एफ
RF	Revolving Fund<br>आर. एफ.
HRF	Health Risk Fund<br>एच. आर. एफ.
FSF	Food Security Fund<br>एफ. एस. एफ.
HF	Health Fund<br>स्वास्थ्य निधि
BL	Bank Loan<br>बैंक लोन या ऋण

CODE FOR Q29. (b)
प्रश्न 29 (ब) के लिए कोड

Item वर्ग	Code कोड
Permanent employment<br>स्थायी रोजगार	1
Seasonal employment<br>मौसमी रोजगार	2
Transfer job<br>स्थानांतरण प्रकृति का काम	3
Natural calamities<br>प्राकृतिक आपदा	4
Community conflicts / riots<br>सामुदायिक संघर्ष/दंगा	5
Animal husbandry<br>पशुपालन	6
Food scarcity at place of residence<br>घर के पास खाद्य सुरक्षा	7
Education and training purpose<br>शिक्षा और प्रशिक्षण के उद्देश्य के लिए	8
Family moved<br>परिवार ने स्थानांतरण किया	9
Other (Specify)<br>उल्लेख करे)	10

Q. NO.	QUESTIONS AND FILTERS प्रश्न और फिल्टर्स	CODING CATEGORIES कोडिंग श्रेणी	SKIP TO पर जाएँ
Q30	What is the religion of the head of the household?<br>घर के मुखिया का धर्म/संप्रदाय क्या है?	HINDU हिंद............ 1<br>MUSLIM मुस्लिम............ 2<br>CHRISTIAN ईसाइ............ 3<br>BUDDHIST/ NEO-BUDDHIST बौद्ध/नव–बौद्ध......... 4<br>SIKH सिक्ख............ 5<br>JAIN जैन............ 6<br>OTHERअन्य ______________ 7<br>**(SPECIFY उल्लेख करे)**	
Q31	What is the caste or tribe of the head of the household?<br>घर के मुखिया की जाति या जनजाति क्या हैं?	CASTE जाति ______________ 1<br>TRIBE जनजाति______________ 2<br>NO CASTE/TRIBE कोई जाति/जनजाति नहीं...... 3<br>DON'T KNOW नहीं पता............ 8	
Q32	**Is this a scheduled caste, a scheduled tribe, other backward class, or general category?**<br>**(IF RESPONDENT NOT REPLIED, CHECK THE LIST OF CASTES/TRIBES AND CODE ACCORDING TO RESPOSE GIVEN IN Q.31)**<br>क्या यह जाति अनुसूचित जाति, अनुसूचित जनजाति या अन्य पिछड़ी जाति या सामान्य जाति है?<br>(यदि प्रतिभागी इसका उत्तर नहीं देता है तो प्रश्न 31 में दिए गए उत्तर के अनुरूप जाति/जनजाति की सूची और कोड देखें)	SCHEDULED CASTE (SC) अनुसूचित जाति................. 1<br>SCHEDULED TRIBE (ST) अनुसूचित जनजाति............. 2<br>OTHER BACKWARD CLASSES (OBC) अन्य पिछड़ी जाति 3<br>GENERAL सामान्य............ 4	
Q33	Do you have ration card?<br>क्या आपके पास राशन कार्ड है?	YES हाँ............ 1<br>NO नहीं............ 0 →	Q36
Q34	**(Ask only if Q33= Yes)**<br>Which type of Ration Card do you have?<br>**यदि प्र. 33 का उत्तर हाँ हो तो –**<br>आपके पास किस प्रकार का राशन कार्ड है?	APLए. पी. एल............ 1<br>BPL बी. पी. एल............ 2<br>ANTYODAYA अन्तयोदय............ 3<br>ANY OTHER कोइ अन्य............ 4	
Q35	PHYSICALLY CHECK AND IDENTIFY THE CARD<br>राशन कार्ड मांगकर और देखकर पहचान करें।	CHECKED देखा............ 1<br>NOT CHECKED नहीं देखा............ 0	
Q36	Does any member of your household have a bank/post office account?<br>आपके परिवार क किसी सदस्य का बैंक या डाकघर में खाता है क्या ?	YES हाँ............ 1<br>NO नहीं............ 0<br>DON'T KNOW नहीं पता............ 8	
Q37	Does your household currently have<br>आपके घर में वर्तमान में क्या–क्या उपलब्ध है<br>A. A MATTRESS चटाई<br>B. A COT OR BED कोट या बिस्तर<br>C. A CHAIR कुर्सी<br>D. A TABLE टेबल<br>E. PRESSURE COOKER प्रेशर कुकर<br>F. ELECTRICITY बिज़ली<br>G. A FAN पंखा<br>H. A RADIO रेडियो	YES हाँ / NO नही<br>A. A MATTRESS चटाई ....1 0<br>B. A COT OR BED कोट या बिस्तर ....1 0<br>C. A CHAIR कुर्सी ....1 0<br>D. A TABLE टेबल ....1 0<br>E. PRESSURE COOKER प्रेशर कुकर ....1 0<br>F. ELECTRICITY बिज़ली ....1 0<br>G. A FAN पंखा ....1 0<br>H. A RADIO रेडियो 1 0	

	I. B/W TELEVISION (TV) ब्लैक एण्ड व्हाइट टी0 वी0<br>J. COLOUR TELEVISION (TV) रंगीन टी0 वी0<br>K. A WATCH OR CLOCK घड़ी<br>L. A TELEPHONE. टेलीफोन<br>M. MOBILE PHONE मोबाईल फोन<br>N. AN ANIMAL-DRIVEN CART बैलगाड़ी/तांगा आदि<br>O. BICYCLE साइकिल<br>P. A MOTORCYCLE मोटरसाइकिल<br>Q. A SEWING MACHINE सिलाई मशीन<br>R. A COMPUTER एक कम्प्यूटर<br>S. INTERNET इंटरनेट<br>T. REFRIGERATOR रेफ्रीज़रेटर<br>U. AN AIR CONDITIONER/COOLER ए.सी./कुलर<br>V. A WASHING MACHINE वाशिंग मशीन<br>W. A WATER PUMP पानी का पंप<br>X. A THRESHER थ्रेशर<br>Y. A TRACTOR ट्रैक्टर<br>Z. A CAR कार<br>AA. GAS CONNECTION (गैस कनेक्शन)<br>BB. GRAIN STORAGE (अनाज भण्डार)	I. B/W TELEVISION (TV) ब्लैक एण्ड व्हाइट टी. वी. ....1 0<br>J. COLOUR TELEVISION (TV) रंगीन टी. वी. ....1 0<br>K. A WATCH OR CLOCK घड़ी ....1 0<br>L. A TELEPHONE. टेलीफोन ....1 0<br>M. MOBILE PHONE मोबाईल फोन..................1 0<br>N. AN ANIMAL-DRIVEN CART बैलगाड़ी/तांगा ....1 0<br>O. BICYCLE साइकिल ....1 0<br>P. A MOTORCYCLE मोटरसाइकिल ....1 0<br>Q. A SEWING MACHINE सिलाई मशीन ....1 0<br>R. A COMPUTER कम्प्यूटर ....1 0<br>S. INTERNET इंटरनेट ....1 0<br>T. REFRIGERATOR रेफ्रीज़रेटर.......................1 0<br>U. AN AIR CONDITIONER/COOLER ए.सी./कूलर...1 0<br>V. A WASHING MACHINE वाशिंग मशीन ....1 0<br>W. A WATER PUMP पानी का पंप ....1 0<br>X. A THRESHER थ्रेशर ....1 0<br>Y. A TRACTOR ट्रैक्टर ....1 0<br>Z. A CAR कार ....1 0<br>AA. GAS CONNECTION (गैस कनेक्शन) ....1 0<br>BB. GRAIN STORAGE (अनाज भण्डार) 1 0	
**Q38**	Do you have any own agricultural land?<br>क्या आपके पास खेती करने की स्वयं की ज़मीन है?	YES हाँ.. 1<br>NO नहीं.. 0	→ **Q40**
**Q39**	**(Ask only if Q38= Yes)**<br>How is the land being cultivated?<br>**(MULTIPLE OPTIONS)**<br><br>**यदि प्रश्न 38 का उत्तर हाँ हो तब ही पूछे –**<br>उस ज़मीन पर खेती कौन करता है?<br>**(बहु विकल्प)**	**YES** **NO**<br>हाँ नहीं<br>A SELF CULTIVATION<br>खुद खेती करते हैं.. 1 0<br>B. SELF BUT GIVEN ON BATAI/LAGAN/ RENT<br>ज़मीन अधिया/किराये/रेग पर दी हुई है.. 1 0<br>C. SELF CULTIVATION and GIVEN ON BATAI/LAGAN/ RENT<br>खुद खेती करते है और ज़मीन अधिया/किराये रेग पर भी दी हुई है..1 0<br>D. NOT USING FOR CULTIVATION.<br>खेती के लिए उपयोग नही करते...........................1 0	
**Q40**	Have you taken any agriculture land on batai / bhaga chasa?<br>क्या आपने खेती के लिए कुछ ज़मीन रेग/अधिया/किराये पर पर ले रखी है?	YES हाँ... 1<br>NO नहीं.. 0	
**Q41**	What kind of toilet facility do members of your household usually use?<br><br>आपके परिवार के सदस्य समान्यता किस	**FLUSH OR POUR FLUSH TOILET**<br>**मल विसर्जन की व्यवस्था या पानी डालकर मल विसर्जित करनेवाले (फ्लश) शौचालय**<br>FLUSH TO PIPED SEWER SYSTEM<br>नाली की पाईप से जुड़ा फ्लश की व्यवस्था..... 01<br>FLUSH TO SEPTIC TANK	

	तरह के शौचालय की सुविधा का उपयोग करते है?	सेप्टिक टैंक में विसर्जित होता है...................... 02<br>FLUSH TO PIT LATRINE<br>पीट शौचालय में मल विसर्जन........................... 03<br>FLUSH TO SOMEWHERE ELSE<br>कहीं और मल विसर्जन..................................... 04<br>FLUSH DON'T KNOW WHERE<br>नहीं पता कि कहाँ मल विसर्जित होता है......... 05<br>**PIT LATRINE पीट** शौचालय<br>PIT VENTILATED IMPROVED<br>हवादार पीट शौचालय .... 06<br>PIT (VIP) BIOGAS LATRINE<br>बायोगैस पीट (VIP) शौचालय........................... 07<br>PIT LATRINE WITH SLAB<br>स्लैब वाला पीट शौचालय .... 08<br>PIT LATRINE WITHOUT SLAB/OPEN PIT<br>खुला/बिना स्लैब वाला पीट शौचालय............ 09<br>TWIN PIT/COMPOSTING TOILET<br>जुड़वा पीट/कंपोस्टिंग शौचालय...................... 10<br>DRY/SERVICE LATRINE<br>सूखा/सेवा शौचालय.. 11<br>NO FACILITY/USES OPEN SPACE OR FIELD/JUNGLE<br>शौचालय की कोई व्यवस्था नहीं है/खुले में या मैदान में या जंगल में शौच करते ह................. 12	
**Q42**	What is the **main** source of drinking water for members of your household?<br>**(RECORD THE SOURCE WHICH IS USED FOR MOST OF THE MONTHS OF THE YEAR)**<br>आपके परिवार के सदस्यों के लिए पीने के पानी का मुख्य स्त्रोत क्या है?<br>(वर्ष के अधिकांश महिने में जिस स्त्रोत का उपयोग किया गया, उसको रिकार्ड करें।)	**PIPE WATER पाईप का पानी**<br>PIPED WATER INTO DWELLING<br>गली के पाईप का पानी.. 01<br>PIPED WATER INTO YARD / PLOT<br>अपनी जमीन में पाईप का पानी.......................... 02<br>PUBLIC TAP / STAND PIPE<br>सार्वजनिक नल/खुला पाईप.............................. 03<br>TUBE WELL OR BOREHOLE<br>हैंडपंप या बोरिंग.. 04<br>**DUG WELL** खुदा हुआ कुआँ<br>PROTECTED DUG WELL सुरक्षित कुआ .... 05<br>UNPROTECTED DUG WELL असुरक्षित कुआ .... 06<br>**WATER FROM SPRING** झरने का पानी<br>PROTECTED SPRING सुरक्षित झरना...................... 07<br>UNPROTECTED SPRING असुरक्षित झरना.............. 08<br>RAINWATER COLLECTION वर्षा के जल का संगह 09<br>TANKER / TRUCK टैंकर/ट्रक................................. 10<br>CART WITH SMALL TANK / DRUM<br>छोटे टंकी/ड्रम वाली गाड़ी................................. 11<br>SURFACE WATER (RIVER, DAM, LAKE, POND, STREAM, CANAL, IRRIGATION CANAL)<br>सतही जल (नदी, बाँध, झील, तालाब, स्ट्रीम, नहर, सिंचाई का नाला).. 12<br>PACKAGED / BOTTLED WATER<br>पैक किया पानी/पानी बोतल.............................. 13<br>COMMUNITY RO PLANT<br>सामुदायिक आर. ओ. प्लांट .... 14<br>OTHER SOURCE अन्य स्त्रोत _______________ 15<br>(**SPECIFY** उल्लेख करे)	

**Q43**	What is the **main** source of water used by your household for other purposes, such as cooking, cloths washing, cleaning of utensils and bathing etc.?<br>**(RECORD THE SOURCE WHICH IS USED FOR MOST OF THE MONTHS OF THE YEAR)**<br>आपके परिवार में अन्य कार्यो जैसे– कपडे धोना, नहाना, खाना बनाना आदि के लिए मुंख्यतः किस स्त्रोत का पानी उपयोग करते है?<br>(**वर्ष के अ धकांश महीने में** जिस पानी के स्त्रोत का उपयोग किया गया, उसे रिकॉर्ड करें।)	**PIPE WATER पाईप का पानी**<br>PIPED WATER INTO DWELLING<br>गली के पाईप का पानी.. 01<br>PIPED WATER INTO YARD / PLOT<br>अपनी जमीन में पाईप का पानी.......................... 02<br>PUBLIC TAP / STAND PIPE<br>सार्वजनिक नल / खुला पाईप.............................. 03<br>TUBE WELL OR BOREHOLE<br>हैंडपंप या बोरिंग.. 04<br>**DUG WELL** खुदा हुआ कुआँ<br>PROTECTED DUG WELL सुरक्षित कुआ.................. 05<br>UNPROTECTED DUG WELL असुरक्षित कुआ.......... 06<br>**WATER FROM SPRING** झरने का पानी<br>PROTECTED SPRING सुरक्षित झरना...................... 07<br>UNPROTECTED SPRING असुरक्षित झरना.............. 08<br>RAINWATER COLLECTION वर्षा के जल का संगह 09<br>TANKER / TRUCK टैंकर / ट्रक................................. 10<br>CART WITH SMALL TANK / DRUM<br>छोटे टंकी / ड्रम वाली गाड़ी................................ 11<br>SURFACE WATER (RIVER, DAM, LAKE, POND, STREAM, CANAL, IRRIGATION CANAL)<br>सतही जल (नदी, बाँध, झील, तालाब, स्ट्रीम, नहर, सिंचाई का नाला)....................................... 12<br>PACKAGED / BOTTLED WATER<br>पैक किया पानी / पानी बोतल............................. 13<br>COMMUNITY RO PLANT<br>सामुदायिक आर. ओ. प्लांट.................................. 14<br>OTHER SOURCE अन्य स्त्रोत _______________ 15<br>(**SPECIFY** उल्लेख करे)	
**Q44a**	Usually people wash/clean hand with different materials. With which material your household members usually wash their hand?<br>सामान्यतः लोग अपना हाथ विभिन्न सामग्रियों के उपयोग से धोते या साफ करते हैं। आपके घर के लोग सामान्यतः किससे हाथ धोते हैं?	BAR SOAP (साबुन)... 1<br>DETERGENT (POWDER/PASTE)<br>डिटर्जेंट पाउडर / पेस्ट.. 2<br>LIQUID SOAP तरल साबुन .... 3<br>ASH / MUD / SAND राख / मिट्टी / रेत......................... 4<br>NONE कुछ नहीं... 5<br>OTHER अन्य________________________________ 6<br>(**SPECIFY** उल्लेख करे)<br>PAIRA पैरा / खड.. 7<br>ONLY WATER सिर्फ पानी.......................................8	

**Q45**	Ask respondent for a teaspoonful of cooking salt currently used and Test Salt for iodine.<br><br>आयोडीन की जाँच के लिए उत्तरदाता से एक चम्मच वर्तमान में उपयोग हो रहे खानेवाल नमक को माँगे और जॉच करे।	00 PPM (No Iodine)<br>00 पी. पी. एम. (आयोडीन नहीं)........................ 0<br>Less than 15 PPM<br>15 पी. पी. एम. से कम...................................... 1<br>More Than 15 PPM<br>15 पी. पी. एम. से अधिक................................. 2<br>NO Salt In Household<br>घर में नमक नहीं है... 3<br>Salt Not tested<br>नमक की जाँच नहीं की गई .... 4<br>**RESULT OF SALT TEST**<br>**नमक जाँच का परिणाम** ☐☐	
**Q46**	Does your household member use soap after defecation?<br>क्या आपके परिवार के सदस्य शौच क बाद हाथ धोने के लिए साबुन का प्रयोग करते हैं?	YES हाँ.. 1<br>NO नहीं... 0	

Q47	(ASK only IF Q.26 = Yes)<br>**In the last one year how many times the SHG related any HH members (member related to Q no 26) have taken a loan?**<br>**If taken then for Same purpose or other?**<br>**यदि प्रश्न 26 हाँ हो तो ही पूछें** – गत 1 वर्ष में घर में बिहान समूह से जुडी किसी महिला सदस्य (**प्रश्न 26 के अनुसार घर का सदस्य का नाम ले**) नें कितनी बार ऋण या लोन लिया है।<br>यदि प्रश्न 47 का उत्तर हाँ हो तो पूछें–घर में बिहान समूह से जुडी किसी महिला सदस्य ने ये ऋण का उपयोग किस उद्देश्य के लिए किया है?

**Sr. No.** क्रं.	**Type of the loan** ऋण का प्रकार	**Number of times loan taken** ऋण कितनी बार लिया गया?	(Yes=1 No=0) **If '0' skip to next option** **Loan used for Same purpose** जिस उद्देश्य के लिए ऋण लिया गया, उसका उपयोग उसी उद्देश्य के लिए किया गया हॉ–1 नही –0 उत्तर यदि 0 या नही है तो अगले कालम में उल्लेख करे	Loan used for other purpose, (Specify) ऋण का उपयोग अन्य उद्देश्य या कार्य के लिए किया–(उल्लेख करें)
**A**	**Community Investment Fund** कम्युनिटी इन्वेस्टमेंट फंड	☐☐	☐	
**B**	**Vulnerability Reduction Fund** वल्नेरेबिलिटी इन्वेस्टमेंट फंड	☐☐	☐	
**C**	**Revolving Fund** रिवाल्विंग फंड	☐☐	☐	
**D**	**Health Risk Fund** हेल्थ रिस्क फंड	☐☐	☐	
**E**	**Food Security Fund** फूड सिक्योरिटी फंड	☐☐	☐	
**F**	**Health Fund** हेल्थ फंड	☐☐	☐	
**G**	**Bank Loan** बैंक लोन	☐☐	☐	

NOTE: SOMETIMES OUR SENIORS/SUPERVISOR MAY COME TO YOU FOR CLARIFICATION OF SOME QUESTION, SO PLEASE COOPERATE WITH THEM.

नोट: कभी–कभी हमारे वरिष्ठ पदाधिकारी/पर्यवेक्षक आपसे कुछ प्रश्नों के बारे में स्पष्टीकरण के लिए आपसे मिलने आ सकते हैं। कृपया उन्हें सहयोग करें।

THANK YOU FOR GIVING YOUR PRECIOUS TIME

अपना बहुमूल्य समय देने के लिए आपका धन्यवाद!

RECORD THE END TIME अंत का समय दर्ज करें:
(In 24 hour format) (24 घंटे के प्रारूप अनुसार) **HOUR घंटा** ☐☐ **MINUTES मिनट** ☐☐
