# Supplementary material for: Context for layering women’s nutrition interventions on a large scale poverty alleviation program: Evidence from three eastern Indian states
Source: PLoS One. 2019 Jan 22;14(1):e0210836. doi: 10.1371/journal.pone.0210836 (PMC6342298; doi:10.1371/journal.pone.0210836)
Supplement: S2 Appendix — Mothers of under two years questionnaire. (PDF) [file pone.0210836.s002.pdf]

SWABHIMAAN
Baseline Survey,2016-17, **Chhattisgarh**
MOTHER OF CHILD UNDER TWO YEARS – QUESTIONNAIRE

स्वाभिमान, बेसलाइन सर्वेक्षण, 2016–17, छत्तीसगढ़

दो वर्ष से कम उम्र के बच्चों की माता की प्रश्नावली

Confidential
For research purpose
शोधकार्य हेतु गोपनीय

A. IDENTIFICATION पहचान

1. STATE राज्य ....

2. DISTRICT जिला ....

3. BLOCK ब्लॉक ....

3.1 AREA (1 = INTERVENTION 2 = CONTROLद्ध क्षेत्र 1– हस्तक्षेप 2 कंट्रोल ....

4. PANCHAYAT पंचायत: ....

5. VILLAGE NAME AND CODE गाँव का नाम एवं कोड____

6. LOCATION OF THE HOUSE (1= MAIN VILLAGE 2= HAMLET) मकान की स्थिति (1–मुख्य गाँव, 2–टोला) ....

6.1 NAME OF HAMLET (IF THE HOUSE IS LOCATED IN THE HAMLET OF VILLAGE)
टोले का नाम (यदि घर गाँव के टोले में अवस्थित है तो)___________________________________

7. HOUSE NUMBER मकान संख्या ....

7.1 SERIAL NUMBER OF UNDER 2 Yrs CHILDREN'S MOTHERS' IN THE VILLAGE..
ग्राम में 2 वर्ष के बच्चे की माता की कम संख्या ---

8. NAME OF THE INVESTIGATOR साक्षात्कारकर्त्ता का नाम____

10. LANDMARK TO LOCATE THE HOUSEHOLD घर की पहचान ____

10.1 NAME OF THE HEAD OF THE HOUSEHOLD घर के मुखिया का नाम ___________________________

11. NAME OF THE MOTHER OF CHILD UNDER TWO बच्चें की माँ का नाम___________________________

11.1 RELATIONSHIP WITH THE HEAD OF THE HOUSEHOLD घर के मुखिया के साथ संबंध_____________

12. UID/ AADHAR CARD NUMBER आधार संख्या ____

13. LINE NUMBER OF THE MOTHER IN HOUSEHOLD QUESTIONNAIRE बच्चें की माँ का परिवार प्रश्नावली में कम संख्या ....

14. SERIAL NUMBER OF MOTHER QUESTIONNAIRE माताओं की प्रश्नावली पत्रक की क्रमांक संख्या---------

B. RESULT STATUS MOTHER QUESTIONNAIRE माताओं की प्रश्नावली पत्रक की परिणाम स्थिति

R1. MOTHER QUESTIONNAIRE माताओं की प्रश्नावली

COMPLETED पूर्ण ---	01
PARTLY COMPLETED आंशिक पूर्ण	02

R2. INTERVIEW DATE साक्षात्कार की तिथि

DATE दिन		MONTH माह		YEAR वर्ष			

R3. NUMBER OF VISITS MADE भ्रमण संख्या

R4. SPOT CHECKED BY (IIPS)___________________________ स्पाट चक (IIPS)]

R5. BACK CHECKED BY (IIPS)___________________________ बैक चैक (IIPS)

R6. FIELD EDITED BY___________________________ फिल्ड एडिट किसके द्वारा

R5. OFFICE EDITED BY___________________________ आफिस एडिट किसके द्वारा

R8. NAME OF THE INVESTIGATOR अनुसंधानकर्त्ता का नाम ____________________

INVESTIGATOR CODE साक्षत्कारकर्ता का कोड

INTRODUCTION AND INFORMED CONSENT

परिचय एवं सूचित सहमति

Namaskar! My name is ____________________________ and I am working with All India Institute of Medical Sciences (AIIMS) in the **"Swabhimaan"** Baseline Project, funded by UNICEF. We are conducting a survey about the health & nutritional status of Adolescent girls (10-19) years, Pregnant Women & Lactating Mothers (mother of child under two years) in the Bastar district of Chhattisgarh state. We would ask you to help us to complete some general questions that are related to your health & nutrition. With your permission, we would like to assess your height, weight and arm circumference; the result of which will also be shared with you and what do those mean at the end of the survey. The information shared by you will remain confidential and would be used only for programme, planning and research purposes. Any personal identifiers that could reveal your identity will be removed before the results of the study are made public or shared between people other than the main researchers working on the project. Your participation in this survey is entirely voluntary and totally based on your willingness; choose not to take part in the survey will not disadvantage you in any way. This interview will take around 25-30 minutes to complete. If you have any questions about the survey feel free to ask me.We thank you for taking time to **understand and showing your interest in the study.**

(परिचय) ''नमस्कार/राम राम/ जोहार'' मेरा नाम __________________________ है । मैं ''स्वाभिमान परियोजना'' में एम्स रायपुर के साथ काम कर रहा/रही हूँ । यह स्वाभिमान परियोजना बस्तर जिले में यूनिसेफ और बिहान के द्वारा चलाई जा रही है। इस परियोजना में हम लोग 10 –19 वर्ष की किशोरियों, गर्भवती महिलाओं और 2 वर्ष की उम्र के बच्चों की माताओं के स्वास्थ्य और पोषण की स्थिति अध्ययन करने के लिए सर्वेक्षण कर रहे हैं।

(उद्देश्य) आज हम स्वाभिमान परियोजना के लिए आपकी स्वीकृति के आधार पर आपके स्वास्थ्य और पोषण की स्थिति से संबंधित प्रश्नों पर बातचीत (साक्षात्कार) करेंगे। साथ ही आपकी स्वीकृति से ही हम आपकी उँचाई, वजन और बाई भुजा की माप को मापना चाहेंगे। बातचीत के अंत में उसकी जानकारी आपको अवश्य बताएँगे।

(गोपनीयता) साक्षात्कार में आपक द्वारा प्रश्नों पर दी गई जानकारियों को गोपनीय रखा जाएगा। इन जानकारियों का उपयोग केवल स्वास्थ्य और पोषण संबंधी कार्यक्रमों, योजनाओं और अध्ययन के लिए किया जाएगा।

आपकी पहचान कभी भी किसी भी स्थिति में प्रकट नही की जाएगी। ऐसी सभी व्यक्तिगत जानकारियां जो आपको इस अध्ययन के प्रतिभागी के रूप में पहचानने में मदद कर सकती हैं, उन्हे किसी अन्य व्यक्ति से साझा करने अथवा प्रकाशित करने से पहले हटा दिए जाएगा।

(पूर्णतः स्वैच्छिक सहभागिता) इस सर्वेक्षण में आपकी सहभागिता आपकी ईच्छा पर आधारित है। इस साक्षात्कार को पूरा करने में 25 से 30 मिनट लगेंगे, जिसमें भाग लेने या ना लेने का निर्णय आप पर है। इसमें भाग लेने या ना लेने से आपको किसी प्रकार का कोई नुकसान नहीं होगा। आपकी भागेदारी स्वैच्छिक है और आप किसी भी समय बिना किसी कारण बताए साक्षात्कार में अपनी सहभागिता के लिए मना कर सकती है।

(उत्तरदाता से सहमति) यदि आप सर्वेक्षण या साक्षात्कार के बारे में कोइ प्रश्न पूछना चाहे तो अवश्य पूछे। और अगर आप सहमत है तो हम आपका साक्षात्कार प्रारंभ कर सकते है।

हम आपका आभार व्यक्त करते हैं कि आपने इसे समझने में समय दिया और इस अध्ययन में अपनी रूचि दिखाई।

VERBAL CONSENT TAKEN मौखिक स्वीकृति ली गई — CONSENT GIVEN स्वीकृति दी गई..............................1

CONSENT NOT GIVEN स्वीकृति नहीं दी गई 2

NAME OF THE RESPONDENT उत्तरदाता का नाम : ___________________________

SIGNATURE OF THE INVESTIGATOR साक्षत्कारकर्ता का हस्ताक्षर : ___________________________

DATE दिनॉक

RECORD THE START TIME साक्षत्कार प्रारंभ करन का समय **HOUR** घंटे ☐☐ **MINUTES** मिनट ☐☐

(In 24 hour format 24 घंटे के फार्मेट में)

Q. No. प्र.सं	QUESTIONS AND FILTERS प्रश्न और फिल्टर्स	CODING CATEGORIES कोडिंग श्रेणी	SKIP TO पर जाएँ
Q01	VERBAL CONSENT TAKEN<br>मौखिक स्वीकृति ली गई।	CONSENT GIVEN स्वीकृति दी गई............................ 1<br>CONSENT NOT GIVEN स्वीकृति नहीं दी गई........... 2<br>WOMAN TEMPORARILY AWAY<br>अभी घर से बाहर गई हुई हैं.............................. 3<br>WOMAN HAS MIGRATED OUT<br>वो महिला प्रवास (बाहर चल गई) पर है........... 4	
Q02	What Is your date of birth?<br>How old were you at your last birthday?<br>आपकी जन्मतिथि क्या हैं?<br>आपके अंतिम जन्मदिन पर आपकी क्या उम्र थी?	DATE OF BIRTH जन्म तिथि [D][D][M][M][Y][Y][Y][Y]<br>AGE IN COMPLETED YEARS उम्र (पूर्ण वर्ष में) [ ][ ]<br>DON'T KNOW नही जानती.......................................98	
Q03	Have you ever attended school/college?<br>क्या आप कभी स्कूल/कालेज गयी हैं?	YES हां .... 1<br>NO नही 0	→ Q 05
Q04	How many years of education have you completed?<br>आपने वर्तमान में कितनी कक्षा तक की पढ़ाई पूरी की है?	YEARS OF EDUCATION COMPLETED<br>**कक्षा/वर्ग (पूरा किया गया)** [ ][ ]	
**STAPLE FOOD AND DIET DIVERSITY<br>मुख्य आहार एवं खाद्य विविधता**			
Q05	What is your family's main staple food?<br>आपके परिवार का मुख्य भोजन (आहार) क्या है?	RICE चॉवल ....1<br>WHEAT गेंहू.. 2<br>MAIZE मक्का .... 3<br>OTHERS अन्य______________________________ 4<br>**(SPECIFY उल्लेख करें)**	
Q06	What is the **main** way that you obtain your family's staple food?<br>**(MULTIPLE OPTIONS)**<br>आपका परिवार अपना मुख्य भोजन (आहार) कहॉ से प्राप्त करता है?<br>(बहु विकल्प संभव)	YES NO<br>A. OWN PRODUCTION खुद उपजाते हैं .... 1 0<br>B. PURCHASED खरीदते हैं .... 1 0<br>C. EXCHANGE FOR LABOUR<br>मज़दूरी के बदले पाते हैं.................................. 1 0<br>D. PDS<br>राशन दुकान 1 0<br>E. OTHERS (BARTER, BORROW etc.) अन्य .... 1 0<br>______________________________<br>**(SPECIFY उल्लेख करें)**	
Q07	Was there any occasion yesterday for which you ate less or more than the usual, like a fast or celebration?<br>कल किसी विशेष अवसर/उत्सव के कारण क्या आपने सामान्य दिनों से कम या अधिक खाना खाया था ? या उपवास के कारण कम या अधिक खाना खाया था ?	YES हाँ.. 1<br>NO नहीं.. 0	1 → Q10

**Q08**	In the last 24 hours, how many times did you eat, including main and small meals during the day and night? (A Meal means consumption of cereal and beverage or cereal 20g alone or milk and milk products or cereal pulse combination. Beverage alone is not considered as a meal.)<br>आपने कल दिन और रात में मिलाकर पिछले 24 घंटे में कुल कितनी बार खाना खाया हैं? मुख्य भोजन और नाश्ते में, घर में या बाहर को मिलाकर बताएँ।<br>(एक आहार का तात्पर्य अनाज आर पेयपदार्थ या 20 ग्राम अनाज दूध या दुग्धउत्पाद के साथ अथवा अनाज–दाल मिश्रण के सेवन से है। पेय पदार्थ को अकेले आहार नहीं माना जाएगा)		DID NOT EAT नहीं खाया .... 1<br>ONCE एक बार .... 2<br>TWICE दो बार.. 3<br>THREE TIMES तीन बार .... 4<br>MORE THAN THREE TIMES तीन बार से अधिक 5	
**Q09**	Now I'd like to ask you about foods and drinks that you ate or drank yesterday during the day or night, whether you ate it at home or anywhere else. I am interested to know whether you had the food items I am going to mention, even if they were combined with other foods.<br>**The investigator needs to read out loud a list of example of food items for each category.**<br>अब मैं आपसे कल दिन–रात मे आपके द्वारा घर पर या बाहर कहीं भी आपके द्वारा खाए या पिए गए भोजन के बारे में पूछना चाहूँगी। आपने जो भी खाया हैं मैं सब कुछ जानना चाहूँगी। यदि आपने खाने की अलग अलग चीजे मिलाकर भी खाया हो तो वह भी जरूर बताएं।<br>**साक्षात्कारकर्त्ता सभी वर्ग के खाद्य–पदार्थों के उदाहरणों को स्पष्ट आवाज़ में सुनाएं।**			
**Q09.1**	**Any foods made from grains, like**<br>**अनाज से बना कोई भोजन, जैसे–**	Wheat, rice, rice flakes, corn, maize, millet or any other grains or foods made from these (e.g. bread, chapati, porridge)<br>गेंहूँ, चावल, टूटा चावल या कनकी, कोदो, मडिया, कुटकी, मक्का, बाज़रा या अन्य अनाज या इनसे बने खाद्य–पदार्थ (उदाहरणार्थ– ब्रेड, रोटी, खिचड़ी)	YES/ हा ....1<br>NO/ नहीं 0	
**Q09.2**	**Any vegetables or roots that are orange-coloured inside, like**<br>**कोई सब्ज़ी या जड़ जो अंदर से नारंगी हो, जैसे–**	Tomato, Pumpkin, carrots, that are yellow or orange inside, jackfruit<br>टमाटर, कुम्हड़ा या कद्दू, गाजर, सब्ज़ी या जड़ जो अंदर से पीली या नारंगी हों।	YES/ हा ....1<br>NO/ नहीं 0	
**Q09.3**	**Any white roots and tubers and plantains like**<br>**कोई सफेद जड़ या कंद या पौधा का भाग जैसे**	White potato, sweet potato, colocasia (arbi), raddish or any other foods made from white-fleshed roots or tubers, or plantains or beetroot<br>सफेद आलू, शकरकंद, अरबी या कोचई, मूली या अन्य भोजन जो उजला भाग वाले जड़ अथवा कंद से बना हो– चुकंदर	YES/ हा ....1<br>NO/ नहीं 0	
**Q09.4**	**Any Medium to Dark green leafy vegetables like:**<br>**कोई हल्की या गहरी हरी पत्तेदार सब्ज़ी**	Methi, spinach/paalak, sarson, , arbi leaves, raddish, beetroot, bathua, Drumstick leaves, Amaranthus, gram leaves, Scallions or Green Onions, Malabar Spinach/ Indian Spinach, Amari Bhaji<br>चरौटा भाजी, मूंगा भाजी, कांदा भाजी, चौलाइ, आमरी, मैथी, पालक भाजी, सरसो भाजी, कोचई पत्ता, मूली भाजी, चुकंदर भाजी, बथुआ भाजी आदि	YES/ हा ....1<br>NO/ नहीं 0	

Q09.5	**Any fruits that are dark yellow or orange inside, like:**<br>**अंदर से हल्का पीला या नारंगी फल, जैसे:**	Ripe mango, Ripe papaya - moosambi, Lemon, Guava, Amla, Bael, Ber<br>पका आम, पका पपीता, नारंगी, मौसम्मी, नींबू, जाम या अमरूद, आँवला, बेर, बेल	YES/ हा ……………………………….1<br>NO/ नहीं ………………………………0	
Q09.6	**Any other fruits like:**<br>**कोई अन्य फल**	Oranges, Singhara, Banana, Apple, Pear, Grapes, Watermelon, Dates, Coconut, Custard Apple, Sapota, Camachile<br>केला, संतरा, तेंदू, गंगा इमली, चार, सेब, नाशपाती, अंगूर, तरबूज, खजूर, नारियल, सीताफल, इमली	YES/ हा ……………………………….1<br>NO/ नहीं ………………………………0	
Q09.7	**Any other vegetables like:**<br>**कोई अन्य सब्ज़ी**	Onion, Brinjal, Cauliflower, Cabbage, Drumstick, Sem, Lauki, Turai, Karela, Ladies Finger, Parwal<br>प्याज़, बैंगन, फूलगोभी, बंदागोभी, मूँगा, सेम, लौकी, तोरई, करेला, भिंडी, परवल	YES/ हा ……………………………….1<br>NO/ नहीं ………………………………0	
Q09.8	**Any meat made from animal organs, such as:**<br>**जनवरों के अंगों का कोई माँस, जैसे:**	Liver, kidney, heart or other organ meats or blood-based foods, including hunted animals<br>अंगो का मांस जैसे कलेजी, पाया, यकृत (लीवर), किड़नी, हृदय या अन्य माँस या खून वाला भोजन, शिकार किये गये जानवर के अंगो का मांस	YES/ हा ……………………………….1<br>NO/ नहीं ………………………………0	
Q09.9	**Any other types of meat or poultry, like:**<br>**किसी अन्य प्रकार का माँस या मुर्गा जैसे:**	Beef, lamb, goat, rabbit, pig, hunted animal's meat, snake, chicken, duck or other bird<br>मुर्गा, बतख, सूअर, मेमना, खरगोश, कोइ शिकार किए जानवर के अंग, साँप, अन्य पक्षी	YES/ हा ……………………………….1<br>NO/ नहीं ………………………………0	
Q09.10	**Any eggs such as:**<br>**कोइ अंडा जैसे**	Eggs from poultry or any other bird<br>मुर्गी का अंडा, बतख का अंडा, किसी चिड़िया का अंडा या अन्य अंडा	YES/ हा ……………………………….1<br>NO/ नहीं ………………………………0	
Q09.11	**Any fish or seafood, whether fresh or dried**<br>**किसी मछली या समुंद्री भोजन, ताज़ा या सुखाया हुआ**	Fresh or Dried Fish, Crabs, Prawns, Shellfish or Seafood<br>केकडा, ताजी या सुखायी हुई मछली, शेलफिश या समुंदी भोजन	YES/ हा ……………………………….1<br>NO/ नहीं ………………………………0	
Q09.12	**Any pulse, Any beans or peas, such as:**<br>**कोई दाल, कोई बिन्स या मटर, जसे:**	Mature Beans or Peas (fresh or dried seed), Lentils (Arhar, Bengal Gram/ Chana, Green Gram/Moog, Black Gram, Moth, Horse Gram /Kulthi) or Bean/Pea products<br>मसूर दाल, अरहर दाल, चना दाल, मूंग दाल, उडद दाल, ताजे या सूखे मटर, ताजे या सूखे छोले,	YES/ हा ……………………………….1<br>NO/ नहीं ………………………………0	

Q09.13	**Any nuts or seeds, like:**<br>**कोई फली या बीज, जैसे :**	Kaju, Badaam, Pista, Cheronjee / Cuddapah Almond, Mungfali/ Groundnut / Peanut or Nut/Seed "Butters" or Pastes<br>चार या चिरौंजी, मूंगफली, मूंगफली का मक्खन, मूंगफली का पेस्ट, काजू, किशमिश, बादाम, पिस्ता	YES/ हा ....1<br>NO/ नहीं 0	
Q09.14	**Any milk or milk products, such as:**<br>**दूध या दूध से बनी चीजे जैसे :**	Milk, cheese, yoghurt or other milk products, but NOT including butter, ice cream, cream or sour cream<br>दूध, पनीर, दही या दूध से बनी चीजे, किंतु मक्खन, आइसक्रीम, मलाई को छोड कर।	YES/ हा ....1<br>NO/ नहीं 0	
Q09.15	**Any Condiments and seasonings such as:**<br>**कुछ मसालेदार या चटपटो चीज जैसे**	Ingredients used in small quantities for flavour, such as chilies, spices, herbs, garlic, fish powder, tomato paste, flavour cubes or seeds, coriander leaves<br>कम मात्रा में स्वाद की चीजे जैसे मिर्च, मसाले, हरी पत्तियाँ, लहसून, मछली पाउडर, टमाटर सॉस, खुशबुदार बीज, धनिया पत्ता आदि	YES/ हा ....1<br>NO/ नहीं 0	
Q09.16	**Other beverages and foods like:**<br>**अन्य पेय पदार्थ एवं भोजन**	Tea or Coffee if not sweetened, clear broth, alcohol, (Drinks of Mahuwa/ Landa/ Salfi/ Chhind), Toddy , Mandia pej<br>फिकी चाय या फिकी कॉफी, छाना हुआ सूप , महुआ की शराब, छिंद रस, ताडी की शराब, सलफी (अलकोहल या शराब)	YES/ हा ....1<br>NO/ नहीं 0	
Q09.17	**Any Insects and other small protein foods such as:**<br>**कोई कीड़ा या प्रौटीन वाला अन्य छोटा भोजन**	Insects, Insect Larvae/Grubs, Insect Eggs and Land and Sea Snails, Red Ant Chutney(Chapra)<br>चापडा, धुला, ज़मीनी घोंगा, समुद्री घोंगा, अन्य कीड़े के अंडे,	YES/ हा ....1<br>NO/ नहीं 0	
Q09.18	**Any Red palm oil**<br>**कुछ लाल ताड़/खजूर का तेल**	Red palm oil<br>लाल ताड़ का तेल	YES/ हा ....1<br>NO/ नहीं 0	
Q09.19	**Any Other oils and fats like:**<br>**अन्य तेल और वसा**	Mustard/ Soybean/ Peanut / Sesame Oil, Fats or Butter added to food or used for cooking, including extracted oils from Nuts, Fruits and Seeds, and all Animal Fat<br>मूंगफली का तेल, सोयाबीन तेल, सरसो तेल, तिल तेल, अन्य फल या बीज का तेल, डालडा, मक्खन, मक्खन से बना हुआ भोजन, सभी प्रकार के जंतु वसा	YES/ हा ....1<br>NO/ नहीं 0	
Q09.20	**Any Savoury and fried snacks such as:**<br>**कुछ लज़ीज या भुना हुआ नाश्ता जैसे**	Crisps and chips, fried dough or other fried snacks<br>चिप्स, पापड़ या अन्य तला हुआ नाश्ता	YES/ हा ....1<br>NO/ नहीं 0	

**Q09.21**	**Any Sweets like:**<br>**कुछ मीठा जैसे**	Sugary foods, such as chocolates, candies, cookies/sweet biscuits & cakes, sweet pastries/ Ice cream, "gur"<br>चीनी/गुड से बनी चीजे जैसे–चॉकलेट, मीठा बिस्कुट, केक, पेस्ट्री,आईसक्रीम, गुड़	YES/ हा ....1<br>NO/ नहीं 0	
**Q09.22**	**Any Sugar-sweetened beverages like:**<br>**चीनी/गुड से बना तरल पेय जैसे**	Sweetened fruit juices and "juice drinks", soft drinks/fizzy drinks, chocolate drinks, malt drinks, yoghurt drinks or sweet tea or coffee with sugar<br>मीठे फल का जूस, जूस ड्रिंक्स, सॉफ्ट ड्रिंक्स, कोक, चॉकलेटी ड्रिंक्स, मीठी लस्सी, मीठा दही, मीठी चाय, मीठी कॉफी, गन्ना रस, मीठा शरबत	YES/ हा ....1<br>NO/ नहीं 0	

FOOD SECURITY – FIES SCALE

खाद्य सुरक्षा – FIES स्तर पर

Household food security - Households are food secure when they have year-round access to the amount and variety of foods required for their members to lead active and healthy lives. At the household level, food security refers to the ability of the household to secure, either from its own production or through purchases, adequate food for meeting the dietary needs of all members of the household.

घरेलु खाद्य सुरक्षा – कोइ घर खाद्य सुरक्षित है यदि इनके सभी सदस्यों के लिए सक्रिय व स्वस्थ जीवन जीने के लिए साल भर के लिए पर्याप्त मात्रा और भिन्न प्रकार के सुरक्षित भोजन पर्याप्त मात्रा में उपलब्ध हैं। घरेलु स्तर पर खाद्य–सुरक्षा होने का तात्पय परिवार के सभी सदस्यों के पोषण आवश्यकताओं की आपूर्ति हेतु स्वतः उत्पादन अथवा खरीदकर खाद्य उपलब्धता सुनिश्चित होने से है।

**Q10**	In last 12 months, was there a time when you were **EVER WORRIED** that you would not be able to get enough food to eat?<br>क्या पिछले 12 माह में कभी ऐसा भी समय आया **आपको फिक्र/चिंता हुई** कि आप या आपका परिवार खाने के लिए पर्याप्त भाजन नहीं प्राप्त कर पाएँगी?	YES हाँ.. 1<br>NO नहीं.. 0	
**Q11**	In last 12 months, was there a time when you were **UNABLE TO EAT HEALTHY AND NUTRITIOUS FOOD** because of a lack of money or other resources?<br>पिछले 12 माह में क्या कभी ऐसा समय आया जब आप या आपका परिवार पैसों की या अन्य संसाधनों की कमी से **अच्छा भोजन** (स्वस्थ और पोषक) **नहीं** कर सका ?	YES हाँ.. 1<br>NO नहीं.. 0	
**Q12**	In last 12 months, was there a time when you **ATE ONLY A FEW KINDS OF FOODS** because of a lack of money or other resources?<br>क्या पिछले 12 माह में कभी ऐसा भी समय आया जब आप पैसों अथवा अन्य संसाधनों की कमी से सिर्फ **एक प्रकार का भोजन ही** कर पाते थे?	YES हाँ.. 1<br>NO नहीं.. 0	
**Q13**	In last 12 months, you **HAD TO SKIP A MEAL** because there was not enough money or other resources to get food?<br>क्या पिछले 12 माह में, कभी भोजन प्राप्त करने के लिए पैसों अथवा अन्य संसाधनों की कमी के कारण कभी आप या आपके परिवार **को खाना छोड़ना पड़ा?**	YES हाँ.. 1<br>NO नहीं.. 0	

**Q14**	In last 12 months, was there a time when you **ATE LESS THAN YOU THOUGHT YOU SHOULD** because of a lack of money or other resources?<br>क्या पिछले 12 माह में पैसों अथवा अन्य संसाधनों की कमी के कारण कभी ऐसा समय आया जब आपने या आपके परिवार ने जितना खाना था उससे कम खाने का सोचा ?	YES हाँ.. 1<br>NO नहीं.. 0	
**Q15**	In last 12 months, was there a time when, your household **RAN OUT OF FOOD** [there was no food at all in household to feed any one] because of a lack of money or other resources?<br>क्या पिछले 12 माह में, कभी ऐसा भी समय आया कि पैसों या अन्य संसाधनों की कमी के कारण आपक घर पर कुछ भी खाने को ना था ? (परिवार के किसी भी सदस्य के लिए घर में कुछ भी खाने के लिए नहीं बचा था)?	YES हाँ.. 1<br>NO नहीं.. 0	
**Q16**	In last 12 months, was there a time when, you were **HUNGRY BUT DID NOT EAT** because there was no food at home and there was not enough money or other resources for food?<br>क्या पिछले 12 माह में ऐसा भी वक्त आया था कि आप भूखी थीं परंतु आपने खाना नही खाया क्योकि घर में भोजन नही था और घर में भोजन या भोजन खरीदने के लिए पैसें भी न थे?	YES हाँ.. 1<br>NO नहीं.. 0	→ **Q17**
**Q16.1**	**(Ask only if Q16=YES)**<br>How often did this happen in the last 12 months?<br>यदि प्रश्न **16** में **हाँ हो तो**<br>गत 12 माहों में ऐसा कितनी बार हुआ?	OFTEN (almost every month) अकसर (लगभग हर माह).. 1<br>SOME TIME (a few months up to 10 months, but not every month) कभी–कभी (10 महीने में एक बार परंतु हर माह नहीं)......................... 2<br>RARELY (once or twice a year) **शायद–संयोग से** (साल में 1 से 2 बार)... 3	
**Q17**	In last 12 months, was there a time when, you did **NOT EAT FOR A WHOLE DAY** because there was no food at home and there was not enough money or other resources for food?<br>क्या पिछले 12 माह में, ऐसा भी समय आया था जब आप घर में खाना नहीं होने के कारण या खाना खरीदने के लिए पैसे नहीं होने के कारण पूरे दिन भूखी रहीं?	YES/ हाँ... 1<br>NO/ नही.. 0<br>DON'T KNOW/ नहीं पता.. 8<br>REFUSED / इनकार किया 9	(0, 8, 9) → **Q18**
**Q17.1**	**(Ask only if Q17=YES)**<br>**How often did this happen in the last 12 months?**<br>यदि, **Q17** में **हाँ हो तो**<br>पिछले 12 माह में ऐसा कितनी बार हुआ?	OFTEN (almost every month) .... 1<br>SOME TIME (a few months up to 10 months, but not every month)... 2<br>RARELY (once or twice a year).............................. 3<br>अक्सर (लगभग हर माह).................................. 1<br>कभी–कभी (10 महीने में एक बार परंतु हर माह नहीं) .... 2<br>शायद–संयोग से (साल में 1 से 2 बार).......... 3	

COPING MECHANISM (The Coping Strategies Index is an indicator of household food security that is relatively simple and quick to use, straight forward to understand, and correlates well with more complex measures of food security. A series of questions about how households manage to cope with a shortfall in food for consumption results in a simple numeric score. The Basic Logic of the CSI is "What do you do when you don't have enough food, and don't have enough money to buy food?") Now I will ask you few questions regarding how you cope up during the food security related problem at house hold level.

सामना करने की प्रक्रिया

(सामना करने की रणनीतियों के सूचक घरेलू खाद्य–सुरक्षा के सूचक होते है। जो तुलनात्मक तौर पर सरल और आसानी से उपयोग किए जा सकते है। इन्हे सरलता से समझा जा सकता है। साथ ही ये खाद्य सुरक्षा के अन्य जटिल मापदण्डों से सीधा संबंध रखते है। घरेलू स्तर पर भोजन की कमी से सामना करने की घरेलू प्रक्रिया से जुड़े प्रश्नों से सरल सांख्यिकी मान प्राप्त किया जाता है। सामना करने की रणनितियों के सूचक (CSI- Coping Strategies Index) इस मूलभूत बात पर आधारित ह कि, ''जब आपके घर में भोजन अथवा भोजन खरीदने के लिए पर्याप्त पैसे नहीं होते हैं तो आप क्या करते हैं ?)

घर में कभी भोजन की कमी होने पर आप या आपका परिवार उसका सामना किस प्रकार करता है, यह जानने के लिए मै आपसे कुछ प्रश्न करूंगी।

**Q18**	HH head now spends extra hours at work to earn more money (overtime)<br>घर में कभी भोजन की कमी होने पर आपके परिवार या घर के मुखिया अधिक आय अर्जित करने के लिए अधिक घंटों तक काम करते है (ओवरटाइम)?	YES हाँ .... 1<br>NO नहीं .... 0<br>DON'T KNOW नहीं पता 8	
**Q19**	Unlike earlier, now female(s) of HH start working outside home<br>घर में कभी भोजन की कमी होने पर परिवार की महिलाएँ भी काम करने बाहर जाने लगती हैं?	YES हा .... 1<br>NO नही .... 0<br>DON'T KNOW नहीं पता 8	(0, 8) → **Q21**
**Q20**	Work that female(s) did for extra income.<br>**(MULTIPLE OPTIONS)**<br>घर की महिलाओं ने अतिरिक्त आय के लिए क्या काम किया?<br>**(कई विकल्प संभव)**	YES NO<br>A. EMBROIDERY कढ़ाई .... 1 0<br>B. TAILORING सिलाई .... 1 0<br>C. SWEEP HOUSE (WORK AS MAID) झाडु–पोंछा .. 1 0<br>D. MAKE SCHOOL BAG LOCKS स्कूल बैग का ताला बनाना .... 1 0<br>E. OTHER अन्य________________ .1 0<br>**(SPECIFY**उल्लेख कर**)**	
**Q21**	Unlike earlier, now children of HH starts working outside home:<br>घर में कभी भोजन की कमी होने पर परिवार के बच्चे भी बाहर काम करने जाने लगते हैं?	YES हाँ .... 1<br>NO नहीं .... 0<br>DON'T KNOW नहीं पता 8	
**Q22**	Migration of a family member to another city to earn money and send it back to the family:<br>घर के सदस्य दूसरे शहर में पैसा कमाने चले जाते हैं और घर पर पैसा भेजते हैं-	YES हाँ .... 1<br>NO नहीं .... 0<br>DON'T KNOW नहीं पता 8	
**Q23**	Borrowing money to meet HH expenses<br>घर के खर्च के लिए कर्ज या उधार लेते है।	YES हाँ .... 1<br>NO नहीं .... 0<br>DON'T KNOW नहीं पता 8	(0, 8) → **Q29**
**Q24**	**(Ask only if Q23=YES)**<br>Money lend from:<br>**(MULTIPLE OPTIONS)**<br>यदि प्रश्न 23 में **हाँ हो तो,** कर्ज या उधार कहाँ से लेते हैं ?<br>(कई विकल्प संभव)	YES हाँ NOनही<br>A. RELATIVES संबंधियों से .... 1 0<br>B. NEIGHBOURS/FRIENDS पड़ोसियों/दोस्तों स .... 1 0<br>C. GROCER किराना दुकान वाले से .... 1 0<br>D. BANKING INSTITUTION बेंक से .... 1 0<br>E. MONEY LENDER सूद/ब्याज पर पैसा देने वालों से .... 1 0<br>F. EMPLOYER मालिक (रोजगारदाता) से .... 1 0<br>G. FROM SHG स्वयंसहायता समूह से 1 0	

**Q25**	Amount of money borrowed (Rs.)<br>कितनो राशि का कर्ज या उधार लिया था? (रूपयों में)	<500500 से कम .... 1<br>>500500 से अधिक.. 2<br>DID NOT DISCLOSE नहीं बताया............................ 3	
**Q26**	Time (duration) for which money borrowed<br>कितनी समयावधि के लिए कर्ज या उधार लिया गया था?	1-2 MONTHS1 – 2 माह .... 1<br>3-5 MONTHS3 – 5 माह .... 2<br>5-12 MONTHS5 – 12 माह..................................... 3<br>12 AND MORE MONTHS12 और अधिक माह.......... 4	
**Q27**	Frequency of borrowing money:<br>एक साल में कितनी बार कर्ज या उधार लिया गया था?	YEARLY 1-2 TIMES साल में 1 – 2 बार.............. 1<br>YEARLY 3-4 TIMES साल में 3 – 4 बार............. 2<br>EVERY MONTH IN YEAR साल में हर माह............ 3<br>DON'T KNOW नहीं जानती.................................... 8	
**Q28**	On an average, monthly Interest (%) charged on borrowed money<br>ली गयी कर्ज या उधार की राशि पर कितना मासिक ब्याज़ लगा?	INTERESTCHARGED………….. ☐☐<br>जिस दर पर ब्याज़ लगा<br>DON'T KNOW/DO NOT REMEMBER……………….98<br>नहीं जानती / याद नहीं है	
**Q29**	Resorted to low-cost food grains/items available<br>क्या आपको गॉव में स्थानीय तौर पर कम दाम के अनाज या अन्य किसी चीज की सहायता मिल पाती है?	YES हाँ .... 1<br>NO नहीं .... 0<br>DON'T KNOW नहीं पता 8	
**Q30**	Borrowing grains to meet food requirements:<br>क्या आपने भोजन आवश्यकताओं के लिए अनाज उधार लिया ?	YES हाँ .... 1<br>NO नहीं .... 0<br>DON'T KNOW नहीं पता 8	
**Q31**	Sold household articles or possessions:<br>क्या आपने घर का सामान या अन्य चीजे बेची ?	YES हाँ .... 1<br>NO नहीं .... 0<br>DON'T KNOW नहीं पता 8	
**Q32**	Coping when there was NO food at home and the family had NO money either to buy food: **(MULTIPLE OPTIONS)**<br>जब घर पर खाना नहीं था और खरीदने के लिए पैसे भी नहीं थे तो ऐसी स्थिति का सामना कैसे किया?<br>**(बहु विकल्प संभव)**	YES NO<br>A. BORROWED FOOD/ खाना या अनाज उधार लिया...1 0<br>B. BORROWED MONEY/ पैसा उधार लिया...........1 0<br>C. SLEPT HUNGRY/ भूखे पेट सो गए............... 1 0<br>D. Other /अन्य___________________________1 0<br>(SPECIFY उल्लेख करें)	
**Q33**	Did village system help you by providing money or grains?<br>क्या गाँव में ऐसी व्यवस्था (अनाज बैंक, धान बैंक या कोष आदि) उपलब्ध है जिससे पैसा या अनाज की सहायता मिलती है?	YES .... 1<br>NO .... 0<br>DON'T KNOW .... 8<br>हाँ.. 1<br>नहीं .... 0<br>नहीं पता... 8	
**Q34**	Did the Jeevika /Village organization help you or your family in any way when you faced such a situation?<br>क्या जब आपने ऐसी परिस्थिति का सामना किया तो बिहान या ग्राम संगठन या महिला स्वयं सहायता समूह ने आपकी या आपके परिवार की किसी प्रकार मदद की?	YES हाँ .... 1<br>NO नहीं .... 0<br>DON'T KNOW नहीं पता 8	0, 8 → **Q36**

**Q35**	**(Ask only if Q34=YES)**<br>In what way?<br>यदि प्रश्न **34** में **हाँ हो तो,**<br>बिहान या **ग्राम** संगठन या महिला स्वयं सहायता समूह किस प्रकार से मदद की?<br>(बहु विकल्प संभव)	YES हाँ / NO नहीं<br>A. PROVIDED GRAINS/FOOD<br>अनाज/भोजन दिया....................................1 0<br>B. PROVIDED MONEY पैसे दिया......................1 0<br>C. OTHER अन्य_____________________________1 0<br>(SPECIFY उल्लेख करें)	
**NUTRITION GARDEN**<br>**बाडी में सब्जी/फल/भाजी/दालें (अर्थात पोषण बगीचा)**			
**Q36**	Does your household have a garden to grow food (Vegetable/ fruits/Pulses) items?<br>क्या आपके घर में सब्जी/फल/भाजी/दाले लगाने (उगाने) के लिए बाडी है?	YES हाँ.. 1<br>NO नहीं.. 0<br>DON'T KNOW नहीं पता... 8	0, 8 → **Q39**
**Q36a**	Since how long you or your household member has planted this nutrition garden?<br>[**Instruction for interviewer:** If response is in week or month convert it into days and write the answer in the provided space.]<br>आप या आपके परिवार के सदस्यों ने घर में सब्जी/फल/भाजी/दाले लगाने (उगाने) की बाडी कब से लगाई है?<br>(इंवेस्टीगेटर ध्यान रखे : यदि उत्तर सप्ताह या माह में हो तो उसे दिन में गणना कर लिखे)	____________Days ago<br>कितने दिनों पहले से सब्जी/फल/भाजी/दाले लगाने (उगाने) की बाडी बनाई गई _______. दिन<br>More than 1 Year 998	
**Q37**	What varieties do you generally grow over a year?<br>**(MULTIPLE OPTIONS)**<br>आप साल भर में अपनी बाडी में समान्यत: क्या–क्या लगा (उपजा) लेती हैं?<br>(बहु विकल्प संभव)	YES हाँ / NO नहीं<br>A. Roots, and tubers - Radish, Carrot, Onion, Arbi, Potato, Beetroot, Garlic<br>जड़, एवं कंद – मूली, गाजर, प्याज, अरबी, आलू, चुकंदर, लहसून................................1 0<br>B. Legumes and nuts – Dates, Mutar, Moong dal, Chana dal, Tur dal<br>दलहन और फली–खजूर, मटर, मूंगदाल, चनादाल..1 0<br>C. Vit A-rich dark green leafy veg Bittle gourd (Lowki), Mustard leaves, Methi, Coriander leaves, Bathua leaves, Spinch, Sanjan Patta<br>विटामिन 'ए' युक्त गहरी हरी पत्तेदार सब्ज़ियाँ, लौकी, सरसो, मैथी धनिया पत्ती, बथुआ साग, पालक, मूंगा पत्ती...1 0<br>D. Other vit A-rich fruits and veg – Papaya, Carrot, water chestnut<br>विटामिन 'ए' युक्त अन्य फल और सब्जियाँ – पपीता, खीरा, तरबूज, शहतूत.............1 0<br>E. Other fruits and vegetables Tomato, Mirch, Sem (braod beans), cauliflower green, Lady finger, Orange, Lal Saag, Banana, Green muttar, Parwal<br>अन्य फल एवं सब्ज़ियाँ – टमाटर, मिर्च सेम, फुलगोभी, भिंडी, नारंगी, लालसाग, केला, हरी मटर, परवल 1 0	

**Q38**	In last seven days, how many days did you consume food (Vegetable/ fruits/Pulses from this garden?<br>पिछले सात दिनों में, आपने अपनी बाडी से कितने दिनो के लिए सब्जियों/फलों/भाजी /दालों का उपयोग खाने के लिए किया है?	NUMBERS OF DAYS FOOD CONSUMED<br>बाडी से सब्जियां/फल/भाजी /दालों के उपयोग दिनों की संख्या ☐	
**PDS AND ICDS SERVICES**<br>**सार्वजनिक वितरण प्रणाली और आई.सी.डी.एस. सेवाएं**			
**Q39**	In the last 30 days or in last one month, did you get a ration from the PDS shop?<br>पिछले 30 दिनों या 1 माह में, क्या आपको राशन दुकान से राशन मिला है?	YES हा... 1<br>NO नहीं... 0<br>DON'T KNOW नहीं पता.. 8	0, 8 → Q41.1
**Q40**	What quantity of Rice (kg) did you receive in the last 30 days or one month from the ration shop?<br>आपको राशन दुकान से पिछले 30 दिनों या 1 माह में कितना चावल (कि.ग्रा.) मिला था?	RICE IN (kg) चावल (कि.ग्रा. में)............. ☐☐<br>DON'T KNOW/ DON'T REMEMBER नहीं पता/याद नहीं 98	
**Q41**	What quantity of wheat/atta (kg) did you receive in the last one month from the ration shop?<br>आपको राशन दुकान से पिछले माह कितना गेंहू/आटा (कि.ग्रा.) मिला था?	WHEAT/ATTA IN (kg) गेहूँ/आटा (कि.ग्रा. में) ☐☐<br>DON'T KNOW/ DON'T REMEMBER नहीं पता/याद नहीं .... 98<br>DIDN'T RECEIVE नही प्राप्त किया 96	
**Q41.1**	Did you ever receive hot cooked meal from AWC?<br>क्या आपने आंगनबाडी केंन्द्र से कभी गर्म भोजन लिया है ?	YES हाँ... 1<br>NO नहीं... 0	
**Q41.2**	Ask only if Q39 is Yes (=1)<br>What quantity of millet (such as Jawar, Bajara, Kodo, Madiya, Ragi, Gram (Chana)) (kg) did you receive in the last one month from the ration shop?<br>आपने पिछले माह राशन दुकान से कितनी मात्रा में कोदो, मडिया, कुटकी, खोसरा बाज़रा (मिलेटस) प्राप्त किया है?	**MILLET IN (kg)** मिलेटस की मात्रा............ ☐☐<br>DON'T KNOW/ DON'T REMEMBER याद नही .... 98<br>**DIDN'T RECEIVE** नही प्राप्त किया...................... **96**	
**Q41.3**	Did you (mother) receive THR from AWC since delivery of (Name of child)?<br>क्या आपने (बच्चे का नाम लें) के प्रसव के बाद से आंगनबाडी केंन्द्र से टेक होम राशन या रेडी टू ईट प्राप्त किया है ?	YES हाँ... 1<br>NO नहीं... 0	
**Q41.4**	Ask only if Q41.3 is Yes (=1)<br>"Do you eat THR at home, received from ICDS?"<br>क्या आप आंगनवाडी (समेकित बाल विकास परियोजना) से प्राप्त टेक होम राशन (रेडी टु इट) स्वंय खाती है?	Only I eat / स्वंय खाती है।.................................1<br>Both I and family members eat / स्वंय भी खाती है और साथ में परिवार के सदस्य भी खाते है.............. 2<br>Only family members eat/ स्वंय नही खाती केवल परिवार के सदस्य खाते है....................3<br>OTHER अन्य________________________8<br>**(SPECIFY** उल्लेख करें**)**	

PREGNANCY AND DELIVERY RELATED QUESTIONS<br>(With your permission, now I would like to talk to you about your last pregnancy, delivery and child)<br>**गर्भावस्था और प्रसव संबंधी प्रश्न**<br>**आपकी अनुमति से अब मैं आपकी पिछली गर्भावस्था, प्रसव एवं बच्चे के बारे में बात करना चाहूँगी।**			
**Q43**	What was the date of your most recent delivery?<br>(बच्चे का नाम लें) का प्रसव (जन्म) कब हुआ था?	DATE OF DELIVERY प्रसव की तिथि D D / M M / Y Y Y Y<br>DON'T KNOW/ DON'T REMEMBER<br>नहीं जानती/याद नहीं 98	
**Q44**	How old the (name) child is?<br>(बच्चे का नाम लें) की उम्र क्या होगी?	AGE IN MONTHSमाह में उम्र	
**Q45**	How many times you have been pregnant, including last pregnancy?<br>आप (बच्चे का नाम लें) के प्रसव को मिलाकर आज तक कुल कितनी बार गर्भवती हुईं हैं?	NO. OF PREGNANCIES<br>कुल गर्भधारण की संख्या	
**Q46**	If you have been pregnant before, how many live birth/still birth and terminations/ miscarriages have you had?<br>आपकी पिछली गर्भावस्थाओं के दौरान कितने जीवित बच्चां का जन्म हुआ? क्या किसी मृत बच्चे ने भी जन्म लिया था? क्या कोई स्वतः गर्भपात भी हुआ था? दवा या डाक्टर के द्वारा बच्चे का गर्भपात कराया गया था क्या?	NUMBER OF LIVE BIRTHS<br>जीवित जन्म की संख्या<br>NUMBER OF STILL BIRTHS<br>मृत जन्म की संख्या<br>NUMBER OF TERMINATED PREGNANCIES (INDUCED ABORTION)<br>डॉक्टर द्वारा कराऐ गए गर्भपात<br>NUMBER OF MISCARRIAGES (SPONTANEOUS ABORTION)<br>स्वतः गर्भपात की संख्या	
**Q47**	What was the interval between your most recent child and the previous childbirth or termination of last pregnancy? (in months)<br>**(ONLY FOR WOMAN WITH TWO OR MORE PREGNANCIES)**<br>**(सिर्फ उन महिलाओं के लिए जो दो या उससे अधिक बार गर्भवती हुई है।)**<br>आपका (बच्चे का नाम लें) और उसके पूर्व के गर्भधारण में कितने समय (माह में) का अंतराल था?	INTERVAL IN MONTHS माह में अंतराल	

**Q48**	Did you use a pregnancy testing kit to know pregnancy status?<br><br>क्या आपने अपने (बच्चे का नाम लें) के समय गर्भवती होने की जाँच के लिए कभी गर्भ जाँच कीट का प्रयोग किया था?	YES हाँ .... 1<br>NO नहीं .... 0<br>DON'T KNOW पता नहीं 8	
**Q49**	Did you register last pregnancy?<br>क्या आपने (बच्चे का नाम लें) की गर्भावस्था दौरान पंजीकरण कराया था	YES हाँ .... 1<br>NO नहीं .... 0<br>DON'T KNOW पता नहीं 8	(0, 8) → **Q53**
**Q50**	How many months pregnant were you when you registered?<br>जब आपने (बच्चे का नाम लें) की गर्भावस्था दौरान पंजीकरण कराया था तो आप कितने माह से गर्भवती थी?	NO. OF MONTHS PREGNANCY REGISTERED<br>पंजीकरण के समय गर्भ का माह [ ]<br><br>DON'T KNOW/ DO NOT REMEMBER<br>पता नहीं/याद नही........ 98	
**Q51**	With whom did you register your pregnancy?<br><br>आपने (बच्चे का नाम लें) की गर्भावस्था दौरान पंजीकरण किनसे कराया था?	ANM एएनएम........ 1<br>ASHA/MITANIN आशा/मितानिन........ 2<br>AWW आँगनबाड़ी कार्यकर्ता........ 3<br>OTHER अन्य__________ ____________________4<br>**(SPECIFY** उल्लेख करें**)**	
**Q52**	Did you receive a Mother and Child Protection Card (MCP) after registration?<br>(See MCP cards)<br>क्या आपको पंजीयन के बाद **मातृ एवं शिशु सुरक्षा कार्ड (एम. सी. पी.)** कार्ड मिला था?<br>**(एम. सी. पी. कार्ड देखें)**	YES हाँ .... 1<br>NO नहीं 0	
**Q53**	Did you see or consult anyone for antenatal care for last pregnancy?<br>[Antenatal Care (ANC) is any care received during pregnancy from any DOCTOR, ANM, MITANIN, AWW or any health provider OR any check-up/test done like laboratory tests, measurement of height, weight, MUAC, blood pressure etc. OR received FA tablets or IFA tablets or Calcium tablets or injection.]<br>क्या आपने गर्भावस्था में किसी से सलाह लो थी या किसी को दिखाया था?<br>(गर्भावस्था दौरान देखभाल से तात्पर्य गर्भावस्था दौरान प्राप्त होने वाली कोई भी सेवा या देखभाल है, जैसे – किसी चिकित्सक, ए.एन.एम, मितानिन, आंगनबाडी कार्यकर्ता या अन्य किसी स्वास्थ्यदाता से प्राप्त सेवा या परामर्श या कोई लैब जांच या परिक्षण या उँचाई मापन, वजन मापन, बांह की गोलाई का मापन या खून जांच, रक्तचाप जांच आदि या आयरन की गोली, कैल्शियम की गोली या कोइ इंजेक्शन प्राप्त करना)	YES हाँ .... 1<br>NO नहीं 0	(0) → **Q73**

**Q54**	Whom did you see or consult?<br>**(MULTIPLE OPTIONS)**<br><br>आपने (बच्चे का नाम लें) की गर्भावस्था दौरान किनको दिखाया या सलाह ली?<br>(बहु विकल्प संभव)	YES हाँ / NO नहीं<br>A. DOCTOR डॉक्टर .... 1 0<br>B. ANMए. एन. एम .... 1 0<br>C. ASHA/MITANIN आशा/मितानिन.................. 1 0<br>D. AWW आँगनबाड़ी कार्यकर्ता .... 1 0<br>E. OTHER अन्य______________________-__1 0<br>**(SPECIFY उल्लेख करें)**	
**Q55**	Where did you receive antenatal care (ANC) for last pregnancy?<br><br>**(MULTIPLE OPTIONS)**<br><br>आपको (बच्चे का नाम लें) की गर्भावस्था दौरान देखभाल की सुविधा कहाँ मिली?<br>**(बहु विकल्प संभव)**	YES हाँ / NO नहीं<br>A. AWC/आँगनबाड़ी केन्द्र .... 1 0<br>B. SC/उप स्वास्थ्य केन्द्र .... 1 0<br>C. PHC प्राथमिक स्वास्थ्य केन्द........................ 1 0<br>D. CHC/सामुदायिक स्वास्थ्य केन्द .... 1 0<br>E. SDH/ CIVIL HOSPITAL सिविल अस्पताल.......... 1 0<br>F. DH /जिला अस्पताल..................................... 1 0<br>G. PVT. HOSPITAL/CLINIC/<br>निजी अस्पताल/क्लिनिक................................ 1 0<br>H. OTHER/अन्य _________________________1 0<br>**(SPECIFY उल्लेख करें)**	
**Q56**	How many months pregnant were you when you first received antenatal check-up (abdominal check-up) for last pregnancy?<br><br>आपकी (बच्चे का नाम लें) की गर्भावस्था दौरान आपकी प्रथम पेट की जाँच (स्वास्थ्य जांच) गर्भावस्था के कौन से माह में हुई थी?	NO. OF MONTHS माह की संख्या [ ]<br><br>DON'T KNOW/DO NOT REMEMBER<br>पता नहीं/याद नही... 98	
**Q57**	How many times did you receive antenatal check-up (abdominal check-up) during last pregnancy?<br><br>(बच्चे का नाम लें) की गर्भावस्था दौरान आपकी पेट की जाँच (गर्भावस्था की देखभाल दौरान) कितनी बार हुई ?	NO. OF TIMES ANC RECEIVED [ ][ ]<br>गर्भावस्था की देखभाल की संख्य<br><br>DON'T KNOW/ DO NOT REMEMBER<br>पता नहीं/याद नहीं ……………….....................98	
**Q58**	Was your weight taken during last pregnancy?<br><br>क्या (बच्चे का नाम लें) की गर्भावस्था दौरान आपका वजन मापा गया था?	YES/ हाँ .... 1<br><br>NO/ नहीं .... 0<br>DON'T KNOW/ नहीं पता...................................... 8	0, 8 → **Q60**

**Q59**	How many times was your weight taken during last pregnancy?<br>(बच्चे का नाम लें) की गर्भावस्था दौरान कितनी बार आपका वजन मापा गया था?	NO.OF TIME WEIGHT TAKEN [ ][ ]<br>वजन माप की संख्या<br>DON'T KNOW/ DO NOT REMEMBER<br>पता नहीं/याद नहीं... 98	
**Q60**	Was your height measured during last pregnancy?<br>क्या (बच्चे का नाम लें) की गर्भावस्था दौरान आपकी उचॉई मापी गयी थी?	YES/ हाँ.. 1<br>NO/ नहीं.. 0<br>DON'T KNOW/ DO NOT REMEMBER<br>नहीं पता/याद नहीं 8	
**Q61**	Was your Mid Upper Arm Circumference (MUAC) measurement taken during last pregnancy?<br>क्या (बच्चे का नाम लें) की गर्भावस्था दौरान आपकी एम.यू.ए. सी. मापी गयी थी?	YES/ हाँ.. 1<br>NO/ नहीं.. 0<br>DON'T KNOW/ DO NOT REMEMBER नहीं पता/याद नहीं 8	
**Q62**	Was your blood pressure measured during last pregnancy?<br>क्या (बच्चे का नाम लें) की गर्भावस्था दौरान आपकी ब्लड प्रेशर (रक्तचाप) मापी गयी थी?	YES/ हाँ.. 1<br>NO/ नहीं.. 0<br>DON'T KNOW/ DO NOT REMEMBER नहीं पता/याद नहीं 8	0, 8 → **Q64**
**Q63**	How many times did your blood pressure measure during last pregnancy?<br>(बच्चे का नाम लें) की गर्भावस्था दौरान कितनी बार आपकी बल्ड प्रेशर (रक्तचाप) मापा गया था ?	NO.OF TIME BP MEASURED [ ][ ]<br>बी. पी. मापों की संख्या ....<br>DON'T KNOW / DO NOT REMEMBER<br>पता नहीं/याद नहीं 98	
**Q64**	Did you have blood test (haemoglobin test) during last pregnancy?<br>(बच्चे का नाम लें) की गर्भावस्था दौरान आपकी हीमोग्लोबिन के लिए खून की जाँच की गयी थी?	YES/ हाँ.. 1<br>NO/ नहीं.. 0<br>DON'T KNOW / DO NOT REMEMBER<br>पता नहीं/याद नहीं 8	
**Q65**	Did you have urine test (Urine strip) during last pregnancy?<br>क्या (बच्चे का नाम लें) की गर्भावस्था दौरान आपके पेशाब की जाँच (यूरीन स्ट्रीप) की गयी थी?(गर्भ जाँच छोडकर)	YES/ हाँ.. 1<br>NO/ नहीं.. 0<br>DON'T KNOW / DO NOT REMEMBER<br>पता नहीं/याद नहीं 8	
**Q66**	During last pregnancy, did you receive TT injection?<br>क्या (बच्चे का नाम लें) की गर्भावस्था दौरान आपको टिटनस की सुई दी गयी थी?	YES/ हाँ.. 1<br>NO/ नहों.. 0<br>DON'T KNOW / DO NOT REMEMBER<br>पता नहीं/याद नहीं 8	0, 8 → **Q67a**

**Q67**	During last pregnancy, how many times did you get a tetanus injection?<br>(बच्चे का नाम लें) की गर्भावस्था दौरान आपको कितनी बार टिटनस की सुई दो गयी थी?	NO.OF TIMES TT INJECTIONS RECEIVED ☐<br>टिटनस सुई की संख्या<br>DON'T KNOW / DO NOT REMEMBER पता नहीं/याद नहीं.. 8	
**Q67a**	During last pregnancy, were you given Folic Acid (F.A.) tablets?<br>(बच्चे का नाम लें) की गर्भावस्था दौरान आपको फॉलिक एसिड (एफ. ए.) की गोली दी गयी थी?	YES हाँ .... 1<br>NO नहीं... 0<br>DON'T KNOW/ DO NOT REMEMBER<br>पता नहीं/याद नहीं... 998	**Q68** (for 0, 998)
**Q67b**	During the whole pregnancy, how many Folic Acid (F.A.) tablets did you receive and consume?<br>(बच्चे का नाम लें) की गर्भावस्था दारान फॉलिक एसिड (एफ. ए.) की कितनी गोली दी गयी थी? और आप ने कितनी गोलीयां खाई थी?	NO. OF FA TABLETS RECEIVED ☐☐☐<br>प्राप्त फॉलिक एसिड (एफ.ए.) गोलियों की संख्या<br>NO. OF FA TABLETS CONSUMED ☐☐☐<br>खाई गयी गोलियों की संख्या<br>DON'T KNOW/ DO NOT REMEMBER<br>पता नहीं/याद नहीं 998	
**Q68**	During last pregnancy, were you given Iron Folic Acid (IFA) tablets?<br>क्या (बच्चे का नाम लें) की गर्भावस्था दौरान आपको आयरन फॉलिक एसिड (आई. एफ. ए.) की गोली दी गयी थी?	YES/ हाँ .... 1<br>NO/ नहीं... 0<br>DON'T KNOW / DO NOT REMEMBER<br>पता नहीं/याद नहीं 8	**Q70** (for 0, 8)
**Q69**	During the whole pregnancy, how many Iron Folic Acid (IFA) tablets did you receive and consume?<br>(बच्चे का नाम लें) की गर्भावस्था दौरान आपको आयरन फॉलिक एसिड (आई.एफ. ए.) की कितनी गोली मिली थी और आपने कितनी गोली खाई थी?	NO.OF IFA TABLET RECEIVED ☐☐☐<br>प्राप्त आयरन फॉलिक एसिड (आई.एफ. ए.)गोलियों की संख्या......<br>NO.OF IFA TABLET CONSUMED ☐☐☐<br>खाई गयी फॉलिक एसिड (आई.एफ. ए.)गोलियों की संख्या<br>DON'T KNOW /DO NOT REMEMBER<br>पता नहीं/याद नहीं 998	
**Q70**	During last pregnancy did you take medicine for deworming?<br>क्या आपने (बच्चे का नाम लें) की गर्भावस्था दौरान कृमिनाशक दवा ली थीं?	YES/ हाँ .... 1<br>NO/ नहीं... 0<br>DON'T KNOW / DO NOT REMEMBER<br>पता नहीं/याद नहीं 8	
**Q71**	During last pregnancy, have you received calcium tablets?<br>क्या आपको (बच्चे का नाम लें) की गर्भावस्था दौरान कैल्शियम की गोली मिली थीं?	YES/ हाँ .... 1<br>NO/ नहीं... 0<br>DON'T KNOW / DO NOT REMEMBER<br>पता नहीं/याद नहीं 8	**Q73** (for 0, 8)
**Q72**	During last pregnancy how many calcium tablets have you taken?<br>आपने (बच्चे का नाम लें) की गर्भावस्था दौरान कैल्शियम की कितनी गोली मिली थीं?	NO.OF CALCIUM TABLETS TAKEN<br>ली गई कैल्शियम की गोलियों की संख्या ☐☐<br>DON'T KNOW / DO NOT REMEMBER<br>पता नहीं/याद नहीं 98	

**Q73**	Did you receive birth preparedness counselling by frontline health worker (ANM, AWW, ASHA/Mitanin)?<br>क्या आपको एएनएम, आंगनवाडी कार्यकर्ता, आशा/मितानिन (गांव स्तर की स्वास्थ्य कार्यकर्त्ताओं) से (बच्चे का नाम लें) के प्रसव की तैयारी के लिए सलाह/परामर्श मिला था?	YES/ हाँ .... 1<br>NO/ नहीं .... 0<br>DON'T KNOW / DO NOT REMEMBER<br>पता नहीं/याद नहीं 8	0, 8 → Q75
**Q74**	What type of counselling did you receive form frontline health worker (ANM, AWW, ASHA/Mitanin)?<br>**(MULTIPLE OPTIONS)**<br>आपको (बच्चे का नाम लें) की गर्भावस्था दौरान एएनएम, आंगनवाडी कार्यकर्ता, आशा/मितानिन (गांव स्तर की स्वास्थ्य कार्यकर्त्ताओं) ने क्या क्या सलाह/परामर्श दिया था?<br>**(बहु विकल्प संभव)**	YES हाँ NO नहीं<br>A. IDENTIFICATION OF PLACE OF DELIVERY<br>प्रसव के स्थान की पहचान .... 1 0<br>B. TRANSPORT FACILITY FOR DELIVERY<br>पसव हेतु परिवहन की सुविधा .... 1 0<br>C. ARRANGEMENT OF MONEY<br>पैसों की व्यवस्था .... 1 0<br>D. BIRTH ATTENDANT<br>प्रसव सहायक .... 1 0<br>E. OTHER/अन्य ________ 1 0	
**Q75**	Have you had any tobacco/alcohol during last pregnancy?<br>क्या आपने कभी इस गर्भावस्था (बच्चे का नाम लें) के दौरान शराब/मादक पदार्थ का सेवन किया था?	YES/ हाँ .... 1<br>NO/ नहीं 0	0 → Q77
**Q76**	Type of tobacco/alcohol you consumed during last pregnancy?<br>**(MULTIPLE OPTIONS)**<br>आपने कभी (बच्चे का नाम लें) की गर्भावस्था दौरान किस प्रकार के शराब/मादक पदार्थ का सेवन किया था?<br>**(बहु विकल्प संभव)**	YES NO<br>A. BIDI/CIGARATE बीड़ी/सिगरेट .... 1 0<br>B. CHEWABLE TOBACCO (KHAINI)<br>चबाने वाला तंबाकु (खैनी) .... 1 0<br>C. GUTKHA गुटखा .... 1 0<br>D. PAN MASALA पान मसाला .... 1 0<br>E. BETEL LEAVES WITH TOBACCO (PAN WITH ZARDA)<br>जर्दावाला पान .... 1 0<br>F. ANY TYPE OF ALCOHOL<br>किसी प्रकार की शराब .... 1 0<br>G. OTHER अन्य ________ 1 0<br>**(SPECIFY** उल्लेख करें**)**	

DELIVERY AND POST NATAL CARE

प्रसव एवं प्रसव पश्चात देखभाल

**Q77**	Was the last delivery normal or caesarean?<br>आपके (बच्चे का नाम लें) का जन्म सामान्य रूप से हुआ था या ऑपरेशन द्वारा?	NORMAL सामान्य .... 1<br>CAESAREAN ऑपरेशन द्वारा 0	
**Q78**	What was the outcome of your recent pregnancy?<br>(बच्चे का नाम लें) की गर्भावस्था दौरान कुल कितने बच्चों का जन्म हुआ था?	SINGLE BIRTH एक.. 1<br>MULTIPLE BIRTHS-TWINS दो............................ 2<br>MULTIPLE BIRTHS-TRIPLETS तीन............................3	
**Q79**	What was the place of last delivery for your most recent pregnancy?<br>(बच्चे का नाम लें) का प्रसव कहाँ हुआ था?	HOMEघर... 1<br>IN TRANSIT अस्पताल जाते समय रास्ते में....... 2<br>GOVERNMENT HEALTH FACILITY<br>सरकारी स्वास्थ्य केन्द्र पर................................. 3<br>PRIVATE HEALTH FACILITY<br>निजी स्वास्थ्य केन्द्र पर...................................... 4	3, 4 → Q81
**Q80**	Who conducted your last delivery?<br>**(In case of home or transit)**<br>**(घर या रास्ते में प्रसव की स्थिति में)**<br>(बच्चे का नाम लें) का प्रसव किसने कराया था?	TBA प्रशिक्षित प्रसव सहायिका........................... 1<br>VILLAGE DOCTOR गाँव का डॉक्टर.................. 2<br>ANM/NURSEए. एन. एम./नर्स........................... 3<br>DOCTOR डॉक्टर .... 4<br>ASHA/MITANIN आशा/मितानिन....................... 5<br>FAMILY MEMBER परिवार की सदस्य................ 6<br>OTHER अन्य ____________________. 7<br>(SPECIFY उल्लेख करे)	
**Q81**	Did anyone advise you for institutional delivery?<br>क्या आपको संस्थागत प्रसव के लिए किसो ने परामर्श दिया था?	YES हाँ .... 1<br>NO नहीं .... 0<br>DON'T KNOW पता नहीं...................................... 8	0, 8 → Q83
**Q82**	Who advised you for institutional delivery?<br>**(MULTIPLE OPTIONS)**<br>आपको संस्थागत प्रसव के लिए किसने परामर्श दिया था?<br>**(बहु विकल्प संभव)**	YES NO / हाँ नहीं<br>A. TBA प्रशिक्षित प्रसव सहायिका.................... 1 0<br>B. VILLAGE DOCTOR (JHOLA CHAP DOCTORS/ BEGA /GUNIA / SIRHA)<br>गाँव का डॉक्टर (झोला छाप डॉक्टर / बैगा / गुनिया / सिरहा)... 1 0<br>C. ANM/NURSEए. एन. एम./नर्स.................... 1 0<br>D. AWW आँगनबाड़ी कार्यकर्ता.......................... 1 0<br>E. DOCTOR डॉक्टर.. 1 0<br>F. ASHA/MITANIN आशा/मितानिन.................. 1 0<br>G. RELATIVES/FRIENDS संबंधी/सहेली.......... 1 0<br>H. JEEVIKA WOMEN GROUP MEMBER<br>बिहान महिला समूह की सदस्या....................... 1 0<br>I. OTHER अन्य______________________________<br>(SPECIFY उल्लेख करे)	

**Q83**	**(Ask only if Q78=1)**<br>Is the last child still alive?<br>यदि, **Q 78 =1 हो तो ही पूछे** –<br>(इनवेस्टीगेटर अभी तक हुइ चर्चा के आधार पर बिना प्रत्यक्ष प्रश्न करे इसका उत्तर लिख सकते है, प्रत्यक्ष प्रश्न टालने का प्रयत्न करे)<br>आपका पिछला बच्चा सकुशल है ?	YES हाँ .... 1<br>NO नहीं 0	
**Q84**	**(Ask only if Q78 =2 or 3)**<br>Are all the children alive now?<br>यदि, **Q 78 = 2 या 3 हो तो ही पूछे** –<br>(इनवेस्टीगेटर अभी तक हुइ चर्चा के आधार पर बिना प्रत्यक्ष प्रश्न करे इसका उत्तर लिख सकते है, प्रत्यक्ष प्रश्न टालने का प्रयत्न करे)<br>आपके सभी बच्चे सकुशल हैं न ?	YES हाँ .... 1<br>NO नहीं 0	
**Q85**	Did you ever breastfeed to your child (name)<br>(बच्चे का नाम लेते हुए पूछें) क्या आपने इसे कभी अपने स्तन का दूध पिलाया हैं?	YES हाँ .... 1<br>NO नहीं .... 0 →	**Q87**
**Q86**	When did you first breastfeed your child?<br>आपने (बच्चे का नाम लें) को पहली बार स्तन का दूध कब पिलाया हैं?	IMMEDIATELY / WITHIN ONE HOUR OF BIRTH<br>जन्म के तुरंत बाद / एक घंटे के अंदर.......... 1<br>1 HOUR TO 4 HOURS 1 से 4 घंटे के अंदर .... 2<br>4 TO 24 HOURS 4 से 24 घंटे के अंदर.............. 3<br>AFTER 24 HOURS 24 घंटे के बाद .... 4<br>DO NOT REMEMBER याद नहीं............................ 5	
**Q87**	Have you given the baby anything other than Breast Milk immediately after the birth such as Cow's Milk/Goat's Milk/ Honey/ Ghutti/ Sugar Water/ Milk Powder/ or any other product?<br>क्या आपने जन्म के बाद अपने शिशु (बच्चे का नाम लें) को स्तन के दूध के अलावा भी कुछ दिया था? जैसे गाय का दूध, भैस का दूध, शहद, घुटटी, शक्कर पानी, दूध पाउडर आदि	YES हाँ .... 1<br>NO नहीं .... 0<br>DON'T KNOW/ DO NOT REMEMBER<br>पता नहीं/याद नहीं.................................. 8	0, 8 → **Q89**
**Q88**	**Ask if Q 78=1 and Q 83= 1 "If 83=0 then skip to 95**<br>What was given?<br>**(MULTIPLE OPTIONS)**<br>यदि, **Q 78 =1** एवं **Q 83 =1 हो तो ही पूछे**<br>**(साथ ही यदि Q 83 =0 हो तो Q 95 पर जाएं)**<br>आपने जन्म के बाद अपने शिशु (बच्चे का नाम लें) को स्तन के दूध के अलावे भी कुछ दिया था ?<br>**बहु विकल्प संभव)**	YES हाँ / NO नहीं<br>A. GOAT'S MILK बकरी का दूध....................... 1 0<br>B. COW'S MILK गाय का दूध......................... 1 0<br>C. HONEY मधु.. 1 0<br>D. GHUTTI जन्मघुट्टी... 1 0<br>E. SUGAR WATER चीनी–पानी......................... 1 0<br>F. MILK POWDER पाउडर का दूध.................. 1 0<br>G. OTHER अन्य________________________ ___1 0<br>(SPECIFY उल्लेख करे)	

**Q89**	Are you currently breastfeeding to the child?<br>क्या आप अभी (बच्चे का नाम लें) को स्तनपान करा रही हैं?	YES हाँ .... 1<br>NO नहीं 0	→ Q92 (if 0)
**Q90**	Was (NAME) breastfed yesterday during the day or at night?<br>(बच्चे का नाम लें) ने कल दिन/रात में स्तनपान किया था?	YES हाँ .... 1<br>NO नहीं 0	
**Q91**	How many times (name) was breastfed in the last 24 hours?<br>कल (बच्चे का नाम लें) ने दिन/रात में कुल कितनी बार स्तनपान किया था?	NUMBER OF TIMES BREASTFED [ ][ ]<br>स्तनपानों की संख्या	
**Q92**	Was (name) given any of these items in the last 24 hours?<br>**(MULTIPLE OPTIONS)**<br>A. PLAIN WATER<br>B. INFANT FORMULA (LOCAL EXAMPLES)<br>C. MILK SUCH AS TINNED, POWDERED, OR FRESH ANIMAL MILK<br>D. JUICE OR JUICE DRINKS<br>E. CLEAR BROTH<br>F. YOGHURT<br>G. THIN PORRIDGE<br>H. ANY OTHER<br>क्या उसे पिछले 24 घंटे में निम्न में से कुछ दिया गया था?<br>**(बहु विकल्प संभव)**<br>सादा पानी<br>नवजात का खाना (स्थानीय उदाहरण)<br>दुध; जेसे–डब्बे का, पाउडर, या गाय/भैंस/बकरी (जानवर) का दूध<br>फल का रस (जूस) या जूस ड्रिंक्स<br>छना हुआ सूप<br>दही<br>पतली खिचड़ी<br>कोई अन्य तरल पदार्थ	YES NO / हाँ नहीं<br>A. PLAIN WATER सादा पानी .... 1 0<br>B. INFANT FORMULA (LOCAL EXAMPLES) नवजात का खाना (स्थानीय उदाहरण) .... 1 0<br>C. MILK SUCH AS TINNED, POWDERED, OR FRESH ANIMAL MILK दुध; जैसे–डब्बे का, पाउडर, या गाय/भैंस/बकरी (जानवर) का दूध .... 1 0<br>D. JUICE OR JUICE DRINKS फल का रस (जूस) या जूस ड्रिंक्स .... 1 0<br>E. CLEAR BROTH छना हुआ सूप .... 1 0<br>F. YOGHURT दही .... 1 0<br>G. THIN PORRIDGE पतली खिचड़ी .... 1 0<br>H. ANY OTHER कोई अन्य तरल पदार्थ ______ 1 0<br>(SPECIFY उल्लेख करे)	
**Q93**	Does (name) normally have any solid or semi-solid foods?<br>क्या उसे कुछ ठोस या अर्द्धठोस भोजन दिया गया था?	YES हाँ .... 1<br>NO नहीं .... 0<br>DON'T KNOW पता नहीं 8	→ Q95 (if 0 or 8)

Q94	How many times did your child receive soft, semi-solid or solid foods other than liquids in the last 24 hours?<br>(do not count small snacks, e.g. 1-2 bites from mothers' or siblings' food)<br>(बच्चे का नाम लें) को पिछले 24 घंटे में कितनी बार तरल पेय पदार्थ के अलावे मुलायम, अर्द्धठोस या ठोस खाना दिया गया?<br>(माँ या भाई–बहनों के साथ 1–2 कौर खाने की गणना न करें)	NO. OF TIME LIQUID/SOFT FOOD<br>तरल/नरम भोजन की संख्या ☐☐<br>NO. OF TIME SEMI SOLID FOOD<br>अर्द्धठोस भोजन की संख्या ☐☐<br>NO. OF TIME SOLID FOOD<br>**ठोस भोजन की संख्या** ☐☐	
Q95	Did you receive IFA tablets after last delivery?<br>क्या आपको (बच्चे का नाम लें) के प्रसव के बाद आई.एफ.ए. की गोली मिली थी?	YES हाँ .... 1<br>NO नहीं........ 0<br>DON'T KNOW पता नहीं 8	
Q96	Did you receive calcium tablets after last delivery?<br>क्या आपको (बच्चे का नाम लें) के प्रसव के उपरांत कैल्शियम की गोली मिली थी?	YES हाँ .... 1<br>NO नहीं........ 0<br>DON'T KNOW पता नहीं 8	
Q97	Did you get benefit/payments from maternity entitlement payments from government?<br>**-Janani Suraksha Yojana (JSY)**<br>क्या आपको शासन की जननी सुरक्षा योजना (जे.एस.वाई.) के तहत मातृत्व लाभ या भुगतान मिला?	NO नहीं........ 1<br>YES हाँ .... 2<br>PAYMENT IS CURRENTLY BEING PROCESSED<br>भुगतान प्रक्रिया में है .... 3<br>UNAWARE/CANNOT REMEMBER<br>मैं नहीं जानती/याद नहीं........ 8	
Q97.1	Did you get benefit/payments from maternity entitlement payments from government?<br>**Indira Gandhi Matritva Sahyog Yojana (IGMSY)/Mamta Yojana**<br>क्या आपको शासन की इंदिरा गॉधी मातृत्व सहयोग योजना (आइजीएमएसवाय.) के तहत मातृत्व लाभ या भुगतान मिला?	NO नहीं........ 1<br>YES हाँ .... 2<br>PAYMENT IS CURRENTLY BEING PROCESSED<br>भुगतान प्रक्रिया में है .... 3<br>UNAWARE/CANNOT REMEMBER<br>मैं नहीं जानती/याद नहीं........ 8<br>........	
Q97.2	Did you get benefit/payments from maternity entitlement payments from government?<br>**Janani Shishu Suraksha Karyakaram (JSSK)**<br>क्या आपको शासन की जननी शिशु सुरक्षा कार्यक्रम (जेएसएसके) के तहत लाभ मिला?	NO नहीं........ 1<br>YES हाँ .... 2<br>PAYMENT IS CURRENTLY BEING PROCESSED<br>भुगतान प्रक्रिया में है .... 3<br>UNAWARE/CANNOT REMEMBER<br>मैं नहीं जानती/याद नहीं........ 8	
Q98	Did you get benefit/payments from maternity entitlement payments from government / taken service of Mahatari Express (102) during your last delivery?" - **Adarsh Dampati Yojna (ADY)/ Mahtari Express**<br>क्या आपको (बच्चे का नाम लें) के प्रसव के समय महतारी एक्सप्रेस (102) का लाभ मिला?	NO नहीं........ 1<br>YES हाँ .... 2<br>PAYMENT IS CURRENTLY BEING PROCESSED<br>भुगतान प्रक्रिया में है .... 3<br>UNAWARE/CANNOT REMEMBER<br>मैं नहीं जानती/याद नहीं........ 8	

**Q99**	Did you receive counselling on complementary feeding by ANM/ Mitanin//AWW on VHNDS?<br>क्या आपको बच्चे के उपरी आहार के बारे में ए.एन.एम / मितानिन / आँगनबाड़ी कार्यकर्ता द्वारा ग्राम स्वास्थ्य, स्वच्छता व पोषण दिवस (वी. एच. एस. एन. डी. – टीकाकरण दिवस) के अवसर पर परामर्श दिया गया था?	YES हाँ .... 1<br>NO नहीं.. 0<br>DON'T KNOW पता नहीं 8	
**Q99a**	Did any frontline health worker (ANM, MITANIN, AWW) (visit your house within 7 days of delivery**? (Ask each one by one)**<br>A. ANM visited within 7 days?<br>B. ASHA/MITANIN visited within 7 days?<br>C. AWW visited within 7days?<br>**(MULTIPLE OPTIONS)**<br><br>क्या ग्राम स्तर के स्वास्थ्य कार्यकर्त्ता (ए. एन.एम / आशा या मितानिन / आँगनबाड़ी) द्वारा प्रसव के 7 दिन के अंदर आपके घर का दौरा किया गया था? (एक–एक कर पूछें)<br>क. क्या ए.एन.एम 7 दिन के अंदर आई थी?<br>ख. क्या आशा या मितानिन 7 दिन के अंदर आई थी?<br>ग. क्या आँगनबाड़ी कार्यकर्ता 7 दिन के अंदर आई थी?<br>(बहु विकल्प संभव)	YES हाँ / NO नहीं / DK पता नहीं<br>A. ANM visited within 7 days. ए.एन.एम 7 दिन के अंदर आइ...... 1 0 8<br>B. ASHA/Mitanin visited within 7days आशा / मितानिन 7 दिन के अंदर आई ...... 1 0 8<br>C. AWW visited within 7 days आँगनबाडो कार्यकर्ता 7 दिन के अंदर आई 1 0 8	
**Q100**	Did any frontline health worker (ANM, AWW, MITANIN (visit your house within 42 days of delivery**? (Ask each one by one)**<br>D. ANM visited within 42 days?<br>E. MITANIN visited within 42 days?<br>F. AWW visited within 42 days?<br>**(MULTIPLE OPTIONS)**<br><br>क्या ग्राम स्तर के स्वास्थ्य कार्यकर्त्ता (ए. एन.एम / आशा या मितानिन / आँगनबाड़ी) द्वारा प्रसव के 42 दिन के अंदर आपके घर का दौरा किया गया था? (एक–एक कर पूछें)<br>क. क्या ए.एन.एम 42 दिन के अंदर आई थी?<br>ख. क्या मितानिन 42 दिन के अंदर आई थी?<br>ग. क्या आँगनबाड़ी कार्यकर्ता 42 दिन के अंदर आई थी?<br>(बहु विकल्प संभव)	YES हाँ / NO नहीं / DK पता नहीं<br>A. ANM visited within 42 days ए.एन.एम 42 दिन के अंदर आइ....... 1 0 8<br>B. ASHA/Mitanin visited within 42 days आशा / मितानिन 42 दिन के अंदर आई ...... 1 0 8<br>C. AWW visited within 42 days आँगनबाड़ी कार्यकर्ता 42 दिन के अंदर आई 1 0 8	

FAMILY PLANNING
परिवार नियोजन

**Q101**	Are you currently doing something or using any method to delay or avoid getting pregnant?<br>क्या आप अभी गर्भधारण को टालने या गर्भधारण में देरी के किसी विधि का प्रयोग कर रही हैं?	YES हाँ .... 1<br>NO नहीं.................... 0<br>DON'T KNOW पता नही 8	0, 8 → **Q 104**
**Q102**	What method do you use?<br>(Respondent may mentioned more than one methods, ask specifically most of the time used methods)<br>आप गर्भधारण को टालने या गर्भधारण में देरी के लिए किसी विधि का उपयोग कर रही हैं?<br>(प्रतिभागी एक से अधिक विधि के बारे में बता सकती हैं; अधिकांशतः उपयोग में लानेवाली विधि के बारे में पूछें।)	A. FEMALE STERILIZATION महिला नसबंदी या बंध्याकरण.................... 01<br>B. MALE STERILIZATION पुरूष नसबंदी.................... 02<br>C. IUD/PPIUD कॉपर टी (आई. यु. डी./पी.पी.आई.यु.डी.................... 03<br>D. INJECTABLES गर्भ निरोधक सुई.................... 04<br>E. PILLS गोली.................... 05<br>F. CONDOM कंडोम.................... 06<br>G. DIAPHRAGM महिला कंडोम (डायफ्राम)....... 07<br>H. STANDARD DAY METHOD सुरक्षित दिन विधि.................... 08<br>I. LACTATION AL AMENORRHOEA METHOD लैक्टेशनल एमिनोरिया विधि.................... 09<br>J. RHYTHM METHOD लय विधि.................... 10<br>K. WITHDRAWAL स्खलनपूर्व निष्कासन .... 11<br>L. OTHER TRADITIONAL METHOD अन्य पारंपरिक विधि.................... 12	08–12 → **Q 104**
**Q103**	From where did/do you or your partner get this method? **(If option A, B, C in question Q102 then only one option C or E will be applicable in this question(103))**<br>**MULTIPLE OPTION**<br>आप अथवा आपके पति कहाँ से इन गर्भनिरोधक उपायों को प्राप्त करते हैं?<br>**(यदि प्रश्न 102 का उत्तर 'क', 'ख' या 'ग' था तो इस प्रश्न 103 में सिर्फ विकल्प 'ग' या 'ड़.' ही लागू होगा)**<br>(बहु विकल्प संभव)	YES हाँ / NO नहीं<br>A. SHOP दुकान.................... 1 0<br>B. PHARMACY दवा दुकान.................... 1 0<br>C. GOVT. CLINIC/HEALTH CENTRE/HOSPITAL सरकारी क्लिनिक/स्वास्थ्य केन्द्र/अस्पताल.. 1 0<br>D. ASHA/MITANIN आशा/मितानिन.................... 1 0<br>E. PVT. DOCTOR प्राइवेट डॉक्टर.................... 1 0<br>F. FRIEND दोस्त/सहेली.................... 1 0<br>E. DON'T KNOW नहीं पता.................... 1 0	
**Q104**	Have you ever visited a health facility, doctor, and nurse, ASHA/MITANIN, ANM or AWW to receive services or information on contraception, pregnancy, abortion or sexually transmitted diseases?<br>क्या आप कभी किसी स्वास्थ्य केन्द्र, चिकित्सक, नर्स, आशा या मितानिन, एएनएम, या आँगनबाड़ी से गर्भनिरोधक, गर्भावस्था, गर्भपात या यौन संचरित रोगों के बारे में जानकारी लेने के लिए मिली हैं?	YES हाँ.................... 1<br>NO नहीं.................... 0	

JEEViKA & AGRICULTURE
बिहान और खेती

**Q105**	Do your family have any agricultural land?<br>क्या आपके परिवार में खेती के लिए ज़मीन है?	YES हाँ.................... 1<br>NO नहीं.................... 0	0 → **Q108**

**Q106**	How much agricultural land does family have?<br>आपके परिवार में खेती की कितनी ज़मीन है?	LAND ज़मीन [ ][ ][ ] . [ ]<br>WRITE UNIT____________________<br>DON'T KNOW/ DON'T REMEMBER<br>पता नही / याद नही 998.9	
**Q107**	What do you or your family grow in that land?<br><br>**(MULTIPLE OPTIONS)**<br>आप या आपके परिवार खेती की ज़मीन में क्या–क्या लगाते / उपजाते हैं?<br>(**बहु विकल्प संभव**)	YES हाँ NO नहीं<br>A. GRAINS/ आनाज .... 1 0<br>B. PULSES/ दालें.. 1 0<br>C. OIL SEEDS/ तिलहन .... 1 0<br>D. VEGETABLES/ सब्ज़ियाँ.................................... 1 0<br>E. Other / अन्य.. 1 0<br>F. Not using/ उपयोग नही करतें.................................. 1 0	
**Q108**	Are you or any member of your household a member of the farmer producer group?<br>क्या आपके परिवार का कोई सदस्य किसान उत्पादक समूह का सदस्य है?	YES हाँ .... 1<br>NO नहीं.. 0<br>DON'T KNOW पता नही 8	
**Q109**	What do the VRP/ KRUSHI MITRA teach you or any member of your household?<br><br>**(MULTIPLE OPTIONS)**<br><br>बिहान कार्यक्रम से जुडे कृषि मित्र आपको अथवा आपके परिवार के किसी सदस्य को क्या क्या सिखाते या बताते हैं?<br><br>**(बहु विकल्प संभव)**	YES NO<br>A. Prepare organic manure using degradable waste of kitchen and / or cow shed and / or agriculture fields<br>रसोईघर या/और गाय के गोबर या/और खेत की जमीन से प्राप्त सडने वाले अपशिष्ट से जैविक खाद बनाना या तैयार करना.. 1 0<br>B. Prepare crop planning for nutrition garden to improve dietary diversity<br>भोजन विविधता सुधारने के लिए घर की बाडी (पोषण बगीचा) के लिए फसल योजना तैयार करना............ 1 0<br><br>C. Prepare organic inputs and follow practices to deal with pest and disease in nutri-farms<br>घर की बाडी (पोषण बगीचा)/खेत को कीडों और बीमारियों से बचाने के लिए जैविक दवा बनाना और उनका उपयाग करना .... 1 0<br>D. Prepare annual calendar for nutri-garden and e) simple methods to improve soil health of nutri-farms<br>घर की बाडी (पोषण बगीचा)/खेत हेतु वार्षिक कैलेण्डर बनाना .... 1 0<br>E. Simple methods to improve soil health of nutri-farms<br>घर की बाडी (पोषण बगीचा)/खेत की मिट्टी के स्वास्थ्य सुधार की सरल विधियां .... 1 0<br>F. DON'T KNOW/ नहीं पता................................... 8	**IF NO, IN ALL OPTIONS THEN GO TO Q111**
**Q110**	Do you or any member of your household feel that the education received from VRP/ KRUSHI MITRA is beneficial?<br>क्या आपको या आपके परिवार को बिहान कार्यक्रम से जुडे कृषि मित्र द्वारा प्राप्त शिक्षा/जानकारी लाभप्रद लगी?	YES हाँ .... 1<br>NO नहीं.. 0<br>DON'T KNOW नहीं पता 8	

Q111	Which practice has you or any member of your household adopted in your farm/ kitchen garden/ any other's farm?<br><br>**(MULTIPLE OPTIONS)**<br>आप या आपका परिवार अपनी बाडी (पोषण बगीचा)/खेत में किस प्रकार की विधियों या तरीके से खेती करता है?<br>(**बहु विकल्प संभव**)	YES हाँ / NO नही<br>A. USE OF BIOLOGICAL INPUTS<br>जैविक इनपुट का उपयोग.............................1 0<br>B. USE OF ORGANIC INPUTS<br>कार्बनिक इनपुट का उपयोग.........................1 0<br>C. INTRODUCE NEW VARITIES OF VEGETABLES<br>सब्ज़ियों की नई प्रजाति को उपजाना.............1 0<br>D. DON'T KNOW नहीं पता 8	
Q112	Do you or any member of your household use pesticide in your farm/ kitchen garden/ any other's farm?<br>क्या आप या आपके परिवार के सदस्य अपनी बाडी (पोषण बगीचा)/खेत में कीटनाशक का प्रयोग/उपयोग करते हैं?	YES हाँ .... 1<br>NO नहीं.. 0<br>DON'T KNOW नहीं पता 8	0, 8 → Q114
Q113	What pesticides you or any member of your household used?<br><br>**(MULTIPLE OPTIONS)**<br>आप या आपके परिवार के सदस्य किस कीटनाशक का प्रयोग/उपयोग करते हैं?<br>(**बहु विकल्प संभव**)	YES हाँ / NO नहीं<br>A. USE OF BIOLOGICAL INPUTS जैविक विधि से निर्मित.......................1 0<br>B. USE OF CHEMICAL रासायनिक...................1 0<br>C. DON'T KNOW नहीं पता 1 0	
Q114	Have you heard of any JEEViKA-led intervention (CGSRLM-BIHAN) in your area?<br>क्या आपने बिहान या आजीविका मिशन से संबंधित कार्या के बारे में सुना है?	YES हाँ .... 1<br>NO नहीं.. 0<br>DON'T KNOW नहीं पता 8	
Q115	Have you attended any meetings about nutrition conducted by the Poshan sakhi/ Mocho Mangun mit (JEEViKA Mobilizer)?<br>क्या आपने पोषण–सखी/मोचोमानगुन मीत (वी.ओ.ए. या बिहान समूह के सदस्य) द्वारा आयोजित किसी पोषण विषय पर चर्चा वाली बैठक में भाग लिया हैं?	YES हाँ .... 1<br>NO नहीं.. 0<br>DON'T KNOW नहीं पता 8	0, 8 → Q119
Q116	How many meetings did you attend in the last 12 months?<br>आपने विगत 12 माह में पोषण–सखी/मोचोमानगुन मीत (वी.ओ.ए. या बिहान समूह के सदस्य) द्वारा आयोजित कितनी बैठकों में भाग लिया है?	NUMBER OF MEETINGS बैठको की संख्या .... |__|__| .... |__|__|	
Q117	How many times did the Poshan Sakhi// Mocho mangun Mit (JEEViKA Mobilizer) visit you at home?<br>पोषण–सखी/मोचोमानगुन मीत (वी.ओ.ए. या बिहान समूह के सदस्य) द्वारा कितनी बार आपके घर भ्रमण किया गया ?	NO. OF TIME POSHAN SAKHI (JEEViKA MOBILIZER) VISITED<br>पोषण–सखी/मोचोमानगुन मीत के भ्रमण की संख्या |__|__|	

**Q118**	What type of counselling did you receive from Poshan Sakhi/Mocho mangun mit (JEEViKA Mobilizer)?<br><br>**(MULTIPLE OPTIONS)**<br><br>पाषण–सखी/मोचोमानगुन मीत (वी.ओ.ए या बिहान समूह के सदस्य) ने आपको किन किन बिन्दुओं पर सलाह/परामर्श दिया है?<br><br>(**बहु विकल्प संभव**)	YES हाँ NO नहीं<br>A. Eating green leafy vegetables after delivery प्रसव के बाद हरी सब्ज़ी खाना..1 0<br>B. Eating more after delivery प्रसव के बाद अधिक बार खाना...1 0<br>C. Exclusive breastfeeding शिशु को केवल स्तनपान ....1 0<br>D. Breastfeeding on demand शिशु की माँग पर स्तनपान...1 0<br><br>E. Continued breastfeeding लगातार स्तनपान...1 0<br>F. Types of immunization needed for the child शिशु के लिए आव" यक टीकाकरण के प्रकार ....1 0<br>G. Feeding frequency (breastfeeding) स्तनपान की आवृत्ति (संख्या)..1 0<br>H. Mother's dietary diversity माता के लिए भोजन विविधता...1 0<br>I. Animal food intake by mother माता को जन्तु से प्राप्य भोजन देना..1 0<br>J. Feeding animal source food to the child शिशु को जन्तु स्त्रोत से प्राप्य भोजन देना ....1 0<br><br>K. Care during child's illness बीमारी के दौरान शिशु की देखभाल...1 0<br>L. Hand washing before food preparationखाना पकाने से पहले हाथ धोना ....1 0<br>M. Hand washing after defecationशौच के बाद हाथ धोना...1 0<br>N. Feeding more during and after child's illness शिशु की बीमारी के दौरान एवं उसके बाद अधिक बार स्तनपान...1 0<br>O. Giving ORS during diarrhoea डायरिया (अतिसार) के दौरान ओ. आर. एस. देना................................1 0<br><br>P. Deworming कृमिनाशक..................................1 0<br>Q. About family planning after birth प्रसव उपरांत परिवार नियोजन..1 0<br>R. About spacing births गर्भ अंतराल के बारे में 1 0<br>S. Help the woman to plan & prepare for birth प्रसव की तैयारी व कार्ययोजना बनाने के लिए..................1 0<br><br>T. Advantages of institutional deliveries संस्थागत प्रसव के लाभ ....1 0<br>U. Advised to take 2 doses of TT injection टिटनेस के दो टीके लेने की सलाह ....1 0<br>V. Advised to take FA, IFA & Calcium tablet supplementation फोलिक ऐसिड, आयरन व कैल्शियम की गोली खाने की सलाह..................................1 0	

		W. Advised to carry out laboratory test (Blood, urine) & anthopometric measurement (Height, weight and MUAC) लैब जांच (खून, पेशाब) व शारीरिक मापन (वजन, उॅचाई, एमयूएसी) करवाने हेतु ……………………………………………………..1 0<br><br>X. Provided information on sex during pregnancy गर्भावस्था दौरान शारीरिक संबंध पर सलाह ...1 0<br>Y. Prepared the women to manage obstetric Complications प्रसव दौरन की जटिलताओं के प्रबंधन पर ……………………………………..................1 0<br>Z. Prepared woman & her family for eventuality in case of an emergency सम्भावित आपात स्थिति हेतु परिवार व महिला को तैयार करना ……………………………..............................1 0<br>AA. Advised to take Nutritional food and avoid heavy work पोष्टिक आहार लेना व भारी काम न करने की सलाह..1 0<br>AB. Advised to take hot cooked meal and THR from AWC आंगनबाडी केन्द्र से नियमित टीएचआर लेना और गर्म पका हुआ भोजन प्राप्त करने की सलाह .... 1 0<br>AC OTHER अन्य______________________1 0<br><br>**(SPECIFY** उल्लेख करें**)**	
**Q119**	In the last three months, has your arm been measured by an Anganwadi worker or a Poshan sakhi /Mocho Mangun mit (Swabhimaan) or members of women's group (JEEViKA Mobilizer) using this kind of tape [show maternal MUAC tape]?<br>क्या विगत 3 माह में कभी, आँगनबाड़ी कार्यकर्ता या पोषण–सखी/मोचोमानगुन मीत (वी.ओ.ए. या बिहान समूह के सदस्य) द्वारा इस प्रकार के टेप से आपके बाजू की माप ली गई है?<br>(माताओं का एम.यु.ए.सी. टेप दिखाऐं)	YES हाँ .... 1<br>NO नहीं.. 0<br>DON'T KNOW नहीं पता 8	
**Q120**	Have you been receiving a double amount of ICDS food?<br>क्या आपको आंगनवाडी (समेकित बाल विकास परियोजना) से दोगुना राशन मिला?	YES हाँ .... 1<br>NO नहीं.. 0<br>DON'T KNOW नहीं पता 8	
**Q121**	Have you attended Village Health Nutrition and Sanitation Day in last 6 months?<br>क्या आपने विगत 6 माह में टीकाकरण दिवस (ग्राम स्वास्थ्य, स्वच्छता और पोषण दिवस) की बैठक में भाग लिया हैं?	YES हाँ .... 1<br>NO नहीं.. 0<br>DON'T KNOW नहीं पता 8	→ **Q123**

**Q122**	How many VHSND days did you attend in the last 6 months?<br>आपने विगत 6 माह में टीकाकरण दिवस (**ग्राम स्वास्थ्य, स्वच्छता और पोषण दिवस**) की कितनी बैठकों में भाग लिया हैं?	NUMBER OF VHSND DAYS **ग्राम स्वास्थ्य, स्वच्छता और पोषण दिवस** की संख्या... |__|__|	
**Q123**	What are the instances in which you use soap to wash your hands?<br>A. After use of toilet<br>B. Before eating<br>C. After attending a child who has defecated<br>D. Before preparing food<br>E. Before feeding a child<br>F. After handling cow dung/dirt<br>**(MULTIPLE OPTIONS)**<br>आप किन अवसरों पर अपन हाथों को धोने के लिए साबुन का उपयोग करती हैं?<br>क. शौचालय उपयोग के बाद<br>ख. खाने से पहले<br>ग. बच्चों का मल साफ करने के बाद<br>घ. खाना पकाने से पहले<br>ड. शिशु को खिलाने से पहले<br>च. गाय का गोबर हटाने या सफाई करने के बाद<br>**(बहु विकल्प संभव)**	YES हाँ / NO नहीं / DK पता नहीं<br>A. After use of toilet शौचालय उपयोग के बाद … 1 0 8<br>B. Before eating खाने से पहले … 1 0 8<br>C. After attending a child who has defecated बच्चों का मल साफ करने के बाद … 1 0 8<br>D. Before preparing food खाना पकाने से पहल … 1 0 8<br>E. Before feeding a child शिशु को खिलाने से पहले … 1 0 8<br>F. After handling cow dung/dirt गाय का गोबर हटाने या सफाई करने के बाद … 1 0 8	
**Q123.1**	How do you dispose off stool of (name)?<br>आप (बच्चे का नाम) के मल का निपटारा किस प्रकार करते हैं?	IN TOILET टॉयलेट में ....1<br>IN DRAIN बहाकर ....2<br>OPEN GARBAGE खुले कचरे में ....3<br>ANY OPEN SPACE अन्य खुली जगह 4	
**ANTHROPOMETRIC MEASUREMENT**<br>**बाजू, उँचाई एवं वजन की माप**			
**Q124**	Can I measure your weight, height and Mid Upper Arm Circumference measurement?<br>क्या मैं आपके बाजू, उँचाई एवं वजन की माप ले सकती हूँ?	YES हाँ .... 1<br>NO नहीं.. 0	→ **Q200**
**Q124.1**<br>**Q124.2**	MUAC (Reading1)<br>एम. यु. ए.सी. 1: (मापन 1)<br>MUAC (Reading2)<br>एम. यु. ए.सी. 2: (मापन 2)<br>**(In Centimetre)**<br>(सेंटीमीटर में)	cm.........................<br>सेंटीमीटर में<br>1 |__|__| . |__| 2 |__|__| . |__|	

**Q124.3**<br>**Q124.4**	Height (Reading1) ऊँचाई : (मापन 1)<br>Height (Reading2) ऊँचाई : (मापन 2)<br><br>**(In Centimetre)**	cm सेंटीमीटर में<br>…………………………<br>1 ___ . _<br>2 ___ . _	
**Q124.5**<br>**Q124.6**	Weight (Reading1) वजन (मापन 1)<br>Weight (Reading2) वजन (मापन 1)<br>**(In kg)**	Kg किग्रा ……………<br>1 ___ . __<br>2 ___ . __	

HUSBAND'S BACKGROUND AND WOMAN'S WORK AND EMPOWERMENT

पति की पृष्टभूमि, महिला के कार्य एवं सशक्तिकरण

**Q200**	What is your marital status?<br><br>क्या आप विवाहित है?<br><br>'	NEVER MARRIED अविवाहित...1<br>CURRENTLY MARRIED विवाहित .... 2<br>REMARRIED पुनःविवाह .... 3<br>WIDOW / WIDOWER विधवा .... 4<br>DIVORCED तलाकशुदा .... 5<br>SEPARATED परित्यक्ता .... 6<br>LIVE-IN-RELATIONSHIP बिना विवाह के साथ रहना .... 7<br>NOT STATED नहीं बताया 8	
**Q200.1**	How old were you when you got married/ started living together?<br><br>आपकी उम्र क्या थी जब आपका विवाह हुआ या आपने साथ रहना प्रारंभ किया?	AGE IN COMPLETED YEARS<br>उम्र पूर्ण वषं में __<br><br>DON'T KNOW पता नहो 98	
**Q 201**	**CHECK Q200 प्रश्न 200 को जांचे**<br><br>**If Q200 = '2' OR '3' OR '7'**<br>यदि **प्रश्न 200 = 2 या 3 या 7 हो तो** ↓<br><br>**If Q200 = '1' OR '8'**<br>यदि **प्रश्न 200 = 1 या 8 हो तो** →<br><br>**If Q200 = '4' OR '5' OR '6'**<br>यदि **प्रश्न 200 = 4 या 5 या 6 हो तो** →		**Q 204**<br><br>**Q 203**
Q202	How old was your husband/ partner on his last birthday?<br><br>आप के पति/सहयोगी की उनके बिते हुए जन्मदिन पर क्या उम्र थी?	AGE IN COMPLETED YEARS __<br><br>उम्र पूर्ण वर्षो में	

**Q203**	Did your (last) husband/partner ever attend school/college? If Yes, what was the highest standard he completed?<br><br>क्या आप के पति/सहयोगी कभी स्कूल या कालेज गए हैं? यदि हाँ तो किस उच्चतम कक्षा तक की पढाई उन्होनें पूर्ण की है?	STANDARD कक्षा. . . . .………… [ ][ ]<br><br>DON'T KNOW नहीं पता . . . . …. . ………….. 98<br>NEVER ATTENDED कभी नहीं गए ……………….....99	
**Q 204**	As you kNOw, some women take up jobs for which they are paid in cash or kind. Others sell things, have a small business or work on the family farm or in the family business. In the last 12 months days, have you done any of these things or any other work?<br>कुछ महिलाएँ काम पर जाती हैं, कुछ महिलाएँ सामान बेचती है, कुछ महिलाएँ छोटा काम–धंधा करती है, या परिवार के खेत में काम करती है या परिवार के किसी काम–धंधे में काम करती है, जिसके लिए महिलाओं को नगद या वस्तु मिलती है।<br>क्या आपने पिछले 12 माह में इस प्रकार का या अन्य कोई प्रकार का कोई काम–धंधा या बनी बूती किया है, जिसके लिए आपको नगद या वस्तु मिली हो ?	YES हाँ . . . . --. . . . . . …..…….1<br><br>NO नहीं . . . . …………... . 0	→ **Q209**
**Q 205**	What is your occupation, that is, what kind of work do you mainly do?<br>आप कौन सा काम–धंधा या बनी बूती करती है? अर्थात किस प्रकार का काम मुख्यतः आप करती हैं जिससे आप को नगद या वस्तु प्राप्त होती है ।	____________________<br><br>____________________	
**Q206**	Do you do this work for a member of your family, for someone else, or are you self-employed?<br>क्या आप यह काम–धंधा या बनी बूती अपने परिवार के सदस्य के लिए, किसी और के लिए या स्वयं के रोजगार के लिए करती हैं?	FOR FAMILY MEMBER परिवार के सदस्यों के लिए 1<br>FOR SOMEONE ELSE किसी और के लिए................2<br>SELF-E MPLOYED स्वयं का रोजगार.........................3	
**Q 207**	Do you usually work throughout the year, or do you work seasonally, or only once in a while?<br>आप सामान्यतः पूरे वर्ष काम–धंधा या बनी बूतो करती हैं या मौसम के अनुसार या केवल एकाद बार	THROUGHOUT THE YEAR पूरे वर्शभर........................1<br>SEASONALLY/PART OF THE YEAR मौसम अनुसार..2<br>ONCE IN A WHILE एकाद बार....................................3	
**Q 208**	Are you paid in cash or kind for this work, or are you NOt paid at all?<br>क्या इस काम–धंधा या बनो बूती के लिए आपको नकद या वस्तु के रूप में भुगतान किया जाता है या इस काम–धंधा या बनी बूती के लिए आपको कभी भुगतान नहीं किया जाता?	CASH ONLY केवल नकद ....1<br>CASH AND KIND नकद व सामग्री ....2<br>IN KIND ONLY केवल सामग्री......................................3<br>NOT PAID भुगतान नहीं किया जाता..........................4	

Q 209	CHECK Q200 प्रश्न 200 को जांचे<br><br>If Q200 = '2' OR '3' OR '7'<br>यदि प्रश्न 200 = 2 या 3 या 7 हो तो ↓<br><br>If Q200 = '1' OR '4' OR '5' OR '6' OR '8'<br>यदि प्रश्न 200 = 1 या 4 या 5 या 6 या 8 हो तो →		Q217
Q 210	CHECK 204 and Q208 प्रश्न 204 एवं 208 को जांचे<br><br>If Q204 = '1' & Q208 = '1' OR '2'<br>यदि प्रश्न 204 = 1 एवं प्रश्न 208 = 1 या 2 हो तो ↓<br><br>If Q204 = '0' OR Q204 = '1' & Q208 = '3' OR '4'<br>यदि प्रश्न 204 = 0 एवं प्रश्न 208 = 3 या 4 हो तो →		Q213
Q 211	Who decides how the money you earn will be used: mainly you, mainly your husband/ partner, or you and your husband/ partner jointly?<br>आपके द्वारा अर्जित की गई आय के उपयोग के संदर्भ में निर्णय कौन लेता है – मुख्यत: आप या मुख्यत: आपके पति/साथी या आप और आपके पति/साथी संयुक्त रूप से ?	RESPONDENT उत्तरदाता ....1<br>HUSBAND/ PARTNER पति/साथी....2<br>RESPONDENT AND HUSBAND/ PARTNER JOINTLY..3<br>उत्तरदाता और पति/साथी संयुक्त रूप से<br>OTHER अन्य 6	
Q 212	Would you say that the money that you earn is more than what your husband/ partner earns, less than what he earns, or about the same?<br><br>क्या आप कहेंगी कि आप अपने पति/साथी की तुलना में –<br>➢ ज्यादा आय अर्जित करती है, या<br>➢ कम आय अर्जित करती है] या<br>➢ उनके बराबर आय अर्जन करती है?	MORE THAN HUSBAND/ PARTNER<br>पति/साथी से ज्यादा ....1<br>LESS THAN HUSBAND/ PARTNER<br>पति/साथी से कम ....2<br>ABOUT THE SAME बराबर ....3<br>HUSBAND/ PARTNER HAS NO EARNINGS<br>पति/साथी की कोई आय नहीं ....4<br>DON'T KNOW पता नहीं 8	

**Q 213**	Who decides how your husband/ partner's earnings will be used: mainly you, mainly your husband/ partner, or you and your husband/ partner jointly?<br>आपके पति/साथी की आय के उपयोग पर निर्णय कौन करता है? मुख्यतः – मुख्यतः आप या मुख्यतः आपके पति/साथी या आप और आपके पति/साथी संयुक्त रूप से ?	RESPONDENT उत्तरदाता ....1<br>HUSBAND/ PARTNER पति/साथी................................2<br>RESPONDENT AND HUSBAND/ PARTNER JOINTLY...........3<br>उत्तरदाता और पति/साथी संयुक्त रूप से<br>SOMEONE ELSE कोई और ....4<br>OTHER अन्य..6	
**Q 214**	Who usually makes decisions about health care for yourself: mainly you, mainly your husband/ partner, you and your husband/ partner jointly, or someone else?<br>आपके स्वास्थ्य की देखभाल का निर्णय सामान्यतः कौन लेता है? मुख्यतः – मुख्यतः आप या मुख्यतः आपके पति/साथी या आप और आपके पति/साथी संयुक्त रूप से ?	RESPONDENT उत्तरदाता ....1<br>HUSBAND/ PARTNER पति/साथी................................2<br>RESPONDENT AND HUSBAND/ PARTNER JOINTLY...........3<br>उत्तरदाता और पति/साथी संयुक्त रूप से<br>SOMEONE ELSE कोई और ....4<br>OTHER अन्य..6	
**Q 215**	Who usually makes decisions about making major household purchases: mainly you, mainly your husband/ partner, you and your husband/ partner jointly, or someone else?<br>घर में की जाने वाली मुख्य खरीददारियों के बारे में सामान्यतः निर्णय कौन करता है? मुख्यतः – मुख्यतः आप या मुख्यतः आपके पति/साथी या आप और आपके पति/साथी संयुक्त रूप से ?	RESPONDENT उत्तरदाता ....1<br>HUSBAND/ PARTNER पति/साथी................................2<br>RESPONDENT AND HUSBAND/ PARTNER JOINTLY...........3<br>उत्तरदाता और पति/साथी संयुक्त रूप से<br>SOMEONE ELSE कोई और ....4<br>OTHER अन्य..6	
**Q 216**	Who usually makes decisions about visits to your family or relatives: mainly you, mainly your husband/ partner, you and your husband/ partner jointly, or someone else?<br>आपके परिवार या रिश्तेदारों के यहाँ जाने का निर्णय सामान्यतः कौन करता है? मुख्यतः – मुख्यतः आप या मुख्यतः आपके पति/साथी या आप और आपके पति/साथी संयुक्त रूप से ?	RESPONDENT उत्तरदाता..1<br>HUSBAND/ PARTNER पति/साथी.................................2<br>RESPONDENT AND HUSBAND/ PARTNER JOINTLY............3<br>उत्तरदाता और पति/साथी संयुक्त रूप से<br>SOMEONE ELSE कोई और ....4<br>OTHER अन्य..6	

**Q 217**	Do you have any money of your own that you alone can decide how to use?<br>क्या आपके पास खुद के पैसे हैं? जिसके उपयोग के बारे में सिर्फ आप अकेले निर्णय कर सकती है ?	YES हाँ...1<br>NO नहीं...0	
**Q 218**	Are you usually allowed to go to the following places alone, only with someone else, or NOt at all?<br>क्या आप सामान्यतः निम्न जगहों पर – अकेले जाते हैं या सिर्फ किसी के साथ या कभी नहीं जाते?<br>a)To the market? बाजार<br>b)To the health facility? स्वास्थ्य केंद्र<br>c)To places outside this (village/ community)? गाँव से बाहर	(sub-table: columns ALONE अकेले / WITH SOMEONE ONLY किसी के साथ ही / NOT AT ALL बिल्कुल नही)<br>A. MARKET बाजार...... : 1 / 2 / 3<br>B. HEALTH FACILITY स्वास्थ्य केंद्र........ : 1 / 2 / 3<br>C. OUTSIDE गाँव से बाहर............. : 1 / 2 / 3	
**Q 219**	Do you have a bank or savings account that you yourself use?<br>क्या आपके पास किसी बैंक का स्वयं का बचत खाता है, जिसका उपयोग आप स्वयं करती हैं?	YES हाँ. . . . . 1<br>NO नहीं. 0	
**Q220**	Do you have any mobile phone that you yourself use?<br>क्या आपके पास कोई मोबाईल फोन है, जिसका उपयोग आप स्वयं करती हैं?	YES हाँ. . . . . 1<br>NO नहीं. 0	
**Q221**	Do you own this or any other house either alone or jointly with someone else?<br>क्या आप यह या अन्य कोई घर का मालिकाना हक रखती हैं ? हॉ तो अकेले या किसी अन्य के साथ संयुक्त तौर पर	ALONE ONLY सिर्फ अकेले. . . . .1<br>JOINTLY ONLY सिर्फ संयुक्त तौर पर . . . . 2<br>BOTH ALONE AND JOINTLY अकेले एवं संयुक्त तौर पर भी . . . . 3<br>DOES NOT OWN कोई घर नहीं 4	
**Q222**	Do you own any land either alone or jointly with someone else?<br>क्या आप किसी जमीन का मालिकाना हक रखती हैं ? हॉ तो अकेले या किसी अन्य के साथ संयुक्त तौर पर ?	ALONE ONLY सिर्फ अकेले. . . . .1<br>JOINTLY ONLY सिर्फ संयुक्त तौर पर . . . . 2<br>BOTH ALONE AND JOINTLY अकेले एवं संयुक्त तौर पर भी . . . . 3<br>DOES NOT OWN कोई घर नहीं 4	
**Q223**	Do you kNOw of any programmes in this area that give loans to women to start or expand a business of their own?<br>क्या आप इस क्षेत्र में ऐसे किसी कार्यक्रम के बारे में जानकारी रखती हैं जो किसी महिला को उसके सवयं के काम–धंधे या उसे बढ़ाने के लिए ऋण देता है?	YES हाँ. . . . . 1<br>NO नहीं. . . . .0 →	**Q225**
**Q224**	Have you yourself ever taken a loan, in cash or in kind, from any of these programmes, to start or expand a business?<br>क्या आपने ऐसे किसी कार्यक्रम से अपना काम–धंधा प्रारंभ करने या उसे बढाने के लिए नकद या अन्य प्रकार से ऋण प्राप्त किया है?	YES हाँ. . . . . 1<br>NO नहीं. 0	
**Q225**	Have you organised or participated in organization of community level programme<br>क्या आपने समुदाय स्तर के किसी कार्यक्रम का आयोजन किया है या उसमें भाग लिया है?	YES हाँ. . . . . 1<br>NO नहीं. 0	

INSTRUCTION: AVOID PRESENCE OF OTHER MEMBERS WHILE ASKING NEXT QUESTION

सूचनाः अगला प्रश्न पूछने के पूर्व किसी अन्य व्यक्ति की उपस्थिति को टालना सुनिश्चित करें

			YES<br>हाँ	NO<br>नहीं	DON'T KNOW<br>पता नहीं	
**Q226**	In your opinion, is a husband/ partner justified in hitting or beating his wife in the following situations:<br>आपके विचार से क्या एक पति/साथी का अपनी पत्नि को मारना या पीटना निम्न परिस्थितियों में उचित है:					**END**<br>समाप्त
	a. If she goes out without telling him?<br>पति/साथी का अपनी पत्नि को मारना या पीटना उचित है: यदि वह उसे बिना कहे बाहर जाती है?	A. GOES OUT . . . . .<br>बाहर जाने पर	1	0	8	
	b. If she neglects the house or the children?<br>पति/साथी का अपनी पत्नि को मारना या पीटना उचित है: यदि वह घर या बच्चों पर ध्यान नहीं देती है?	B. NEGL. CHILDREN<br>बच्चों पर ध्यान नहीं देना	1	0	8	
	c. If she argues with him?<br>पति/साथी का अपनी पत्नि को मारना या पीटना उचित है: यदि वह उससे बहस करती है?	C. ARGUES . . . .<br>बहस करने पर	1	0	8	
	d. If she refuses to have sex with him?<br>पति/साथी का अपनी पत्नि को मारना या पीटना उचित है: यदि वह उसे शारीरिक संबंध के लिए मना करती है?	D. REFUSES SEX. .<br>शारीरिक संबंध के लिए मना	1	0	8	
	e. If she doesn't cook food properly?<br>पति/साथी का अपनी पत्नि को मारना या पीटना उचित है: यदि वह ठीक ढंग से खाना नहीं पकाती है?	E. POOR COOKING<br>खराब खाना	1	0	8	
	f. If he suspects her of being unfaithful?<br>पति/साथी का अपनी पत्नि को मारना या पीटना उचित है: यदि वह उस पर धोखा देने का शक करता है?	F. UNFAITHFUL. .<br>धोखा देना	1	0	8	
	g. If she shows disrespect for in-laws?<br>पति/साथी का अपनी पत्नि को मारना या पीटना उचित है: यदि वह सास के लिए असम्मान प्रदर्शित करे?	G. DISRESPECT . . .<br>असम्मान	1	0	8	

NOTE: SOMETIMES OUR SENIORS/SUPERVISOR MAY COME TO YOU FOR CLARIFICATION OF SOME QUESTION, SO PLEASE COOPERATE WITH THEM.

नोटः कभी–कभी हमारे वरिष्ठ पदाधिकारी/पर्यवेक्षक आपसे कुछ प्रश्नों के बारे में स्पष्टीकरण के लिए आपसे मिलने आ सकते हैं। कृपया उन्हें सहयोग करें।

THANK YOU FOR GIVING YOUR PRECIOUS TIME

अपना बहुमूल्य समय देने के लिए आपका धन्यवाद!

RECORD THE END TIME: HOUR घंटा ☐☐ **MINUTES** मिनट ☐☐

अंत का समय दर्ज करें

(In 24 hour format)
